# Bidirectional Modulation of the Claustrum–Postrhinal Cortex Circuit Alleviates Motion Sickness in Mice: A Cortical Target for Sensory Conflict Resolution

**DOI:** 10.1002/cns.71261

**Published:** 2026-11-12

**Authors:** Wen‐Rui Jiang, Xin‐Tong Qiu, Zi‐Yi Yang, Yu‐Tong Guo, Zi‐Yan Wang, Jia‐Rui Xu, Lu Yang, Zhong‐Xuan Du, Xiao‐Tian Yang, Tian‐Yu Zhao, Fu‐Xing Zhang, Rui‐Chao Gou, Pan Wang, Ming‐Ming Zhang

**Affiliations:** ^1^ Department of Anatomy and K.K. Leung Brain Research Centre, School of Basic Medicine The Fourth Military Medical University Xi'an China; ^2^ Educational Testing Evaluation Center The Fourth Military Medical University Xi'an China

**Keywords:** claustrum, gait analysis, motion sickness, postrhinal cortex, spatial orientation

## Abstract

**Background:**

Motion sickness (MS) is not merely a vestibular‐autonomic reflex disorder, but rather a neurological condition involving pathological disturbances in higher cortical functions. Currently, there is a lack of in‐depth understanding of its underlying cortical mechanisms.

**Methods:**

Using a patented double‐axis rotation apparatus combined with Morris water maze and CatWalk gait analysis, we investigated the role of the claustrum–postrhinal cortex (CLA–POR) circuit in a mouse model of MS.

**Results:**

MS suppressed neuronal activity in both CLA and POR, as evidenced by reduced FOS expression. Pathway‐level analysis revealed that MS altered the excitatory‐inhibitory (E/I) balance in CLA–POR projections, decreasing glutamatergic and increasing GABAergic neuron activity. Chemogenetic activation of CLA^VGluT2^–POR or inhibition of CLA^GAD67^–POR projections both alleviated MS, whereas opposite manipulations produced no significant behavioral changes in either MS or healthy mice.

**Conclusion:**

These findings establish the CLA–POR circuit as a bidirectional regulator of MS pathophysiology, where the E/I balance governs spatial orientation, offering a novel cortical target for therapeutic intervention.

## Graphical abstract

Double‐axis rotation induces motion sickness (MS) and shifts the claustrum‐postrhinal cortex (CLA–POR) excitatory‐inhibitory (E/I) balance. This sensory conflict remodels the activity of excitatory and inhibitory neuronal populations within the CLA. Chemogenetic restoration of this balance alleviates spatial disorientation and gait abnormalities, identifying a novel cortical therapeutic target for MS.

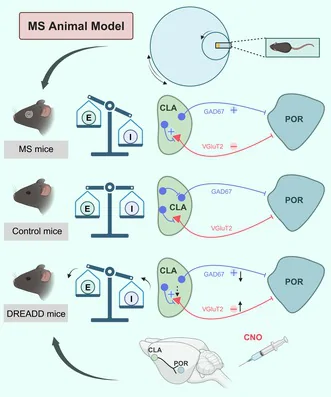

## 1 Introduction

Motion sickness (MS) is a common yet complex disorder arising from conflicting sensory inputs among the vestibular, visual, and proprioceptive systems. While MS is clinically characterized by autonomic symptoms including nausea, vomiting, pica, and defecation, its most functionally debilitating features are severe spatial disorientation and impaired motor coordination, which compromise navigation capabilities and operational performance in affected individuals [1, 2]. Despite the substantial socioeconomic burden imposed by these sensorimotor impairments, no standardized, quantifiable metrics for spatial disorientation or motor dysfunction have been established in experimental animal models, severely limiting mechanistic investigation and therapeutic development. Therefore, elucidating the neural substrates underlying MS‐related spatial and motor deficits, and establishing reproducible behavioral paradigms for their assessment, represents an essential priority for both basic research and clinical translation.

Despite extensive research, the central pathogenesis of MS remains incompletely understood. Currently, the neural mismatch theory is considered the central theory explaining the onset of MS [3, 4]. Numerous previous studies have shown that the vestibular system, as a key structure mediating spatial orientation and postural balance, is a critical region for perceiving kinesthetic signals. In recent years, the involvement of the cerebral cortex in MS has received increasing attention. However, most studies have focused on regions such as the medial prefrontal cortex (mPFC), while less attention has been paid to the claustrum (CLA) and the postrhinal cortex (POR), two brain regions involved in higher‐order central functions [5, 6]. The CLA is one of the most extensively connected brain regions per unit volume and is involved in the regulation of multiple higher‐order brain functions, including consciousness, attention, and multisensory integration [7, 8]. Its dense projections to the POR, a key region for spatial memory encoding, position the CLA–POR pathway as a potential cortical hub where multisensory conflict induces pathological circuit remodeling [9, 10]. However, whether this pathway regulates MS‐induced spatial disorientation remains unknown.

The present study was designed to address these research gaps. We used a double‐axis rotation model to elicit MS responses and spatial disorientation in rodents [11]. By integrating whole‐brain FOS immunofluorescence morphology with a series of behavioral assays, including the Morris water maze test and gait analysis, we established a systematic evaluation system in mice to enable quantitative assessment of both MS symptoms and spatial functional deficits. Finally, using neural circuit tracing, chemogenetic manipulation, and behavioral validation, we systematically dissected the regulatory role of the CLA–POR neural circuit in MS. This study provides new perspectives for understanding the higher‐order central regulatory mechanisms of MS and offers novel insights into the role of the CLA–POR pathway in MS‐induced spatial dysfunction.

## 2 Materials and Methods

### 2.1 Animals

Six to eight‐week‐old C57BL/6J, *vesicular GABA transporter (VGAT)‐Cre*, and *vesicular glutamate transporter 2 (VGluT2)‐Cre* mice were provided by the Experimental Animal Center of Fourth Military Medical University. Animals were housed in a standardized environment under a 12‐h light/dark cycle (lights on from 8:00 to 20:00). All procedures complied with institutional and national guidelines and were approved by the Animal Care and Use Committee of the Fourth Military Medical University (IACUC‐20211018). All behavioral experiments, including MS induction, open field test (OFT), Morris water maze, and CatWalk analyses, were conducted during the light phase within a fixed daily time window (12:00–17:00) to minimize circadian confounding.

Sex as a biological variable. Both male and female mice were included in the initial behavioral phenotyping experiments (Figures 1, S2, and S3). Because female mice exhibited no significant spatial or motor deficits under the present stimulation paradigm (see Section 3.1), all subsequent circuit‐level experiments were performed in male mice. This single‐sex design was adopted to reduce animal use and inter‐individual variance; the potential mechanisms underlying this sexual dimorphism are addressed in the Discussion as an important direction for future investigation.

**FIGURE 1:**
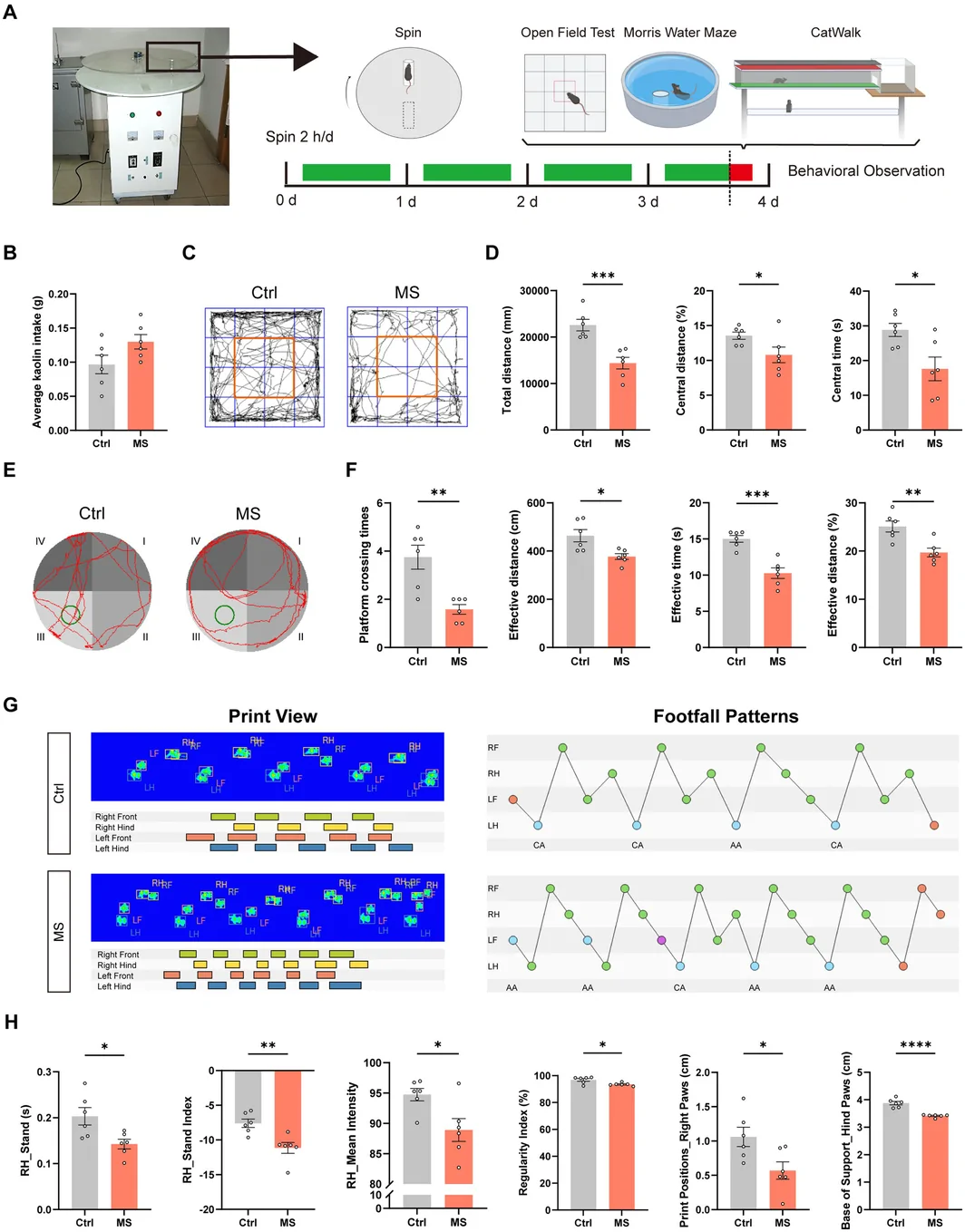
Establishment of motion sickness (MS) model by double‐axis rotation apparatus. (A) Experimental timeline. (B) Average kaolin intake in mice (*n* = 6 mice per group). (C) Representative activity track maps of spontaneous activity from the indicated groups. (D) Total distance, central distance (%), and central time for open field test (OFT) in mice (*n* = 6 mice per group). (E) Representative activity track maps of Morris water maze from the indicated groups. (F) Platform crossing times, effective distance, effective time, and effective distance (%) for Morris water maze in mice (*n* = 6 mice per group). (G) The print view and footfall patterns of the step sequence in a mouse that passes through the glass pane. Red circle, not taken into account. Blue circle, start of a pattern. Green circle, part of a pattern. Purple circle, not part of a pattern. CA, cruciate pattern (right front [RF]–left front [LF]–right hind [RH]–left hind [LH]). AA, alternate pattern (RF–RH–LF–LH). (H) RH stand, RH stand index, RH mean intensity, Regularity index, Print Positions right paws, and Base of Support hind paws for CatWalk gait analysis in mice (*n* = 6 mice per group). Data are mean ± SEM; statistical analyses were conducted using unpaired *t*‐test; *p < 0.05, **p < 0.01, ***p < 0.001, ****p < 0.0001. Statistical details are provided in Table S1. Ctrl, control; LF, left front; LH, left hind; MS, motion sickness; RF, right front; RH, right hind.

### 2.2 Drugs

In the chemogenetic experiment, mice were *i.p*. administered 3 mg/kg clozapine N‐oxide (CNO, C0832, Sigma‐Aldrich, St. Louis, MI, USA) or 50 μL saline at 4 weeks following virus injection.

### 2.3 Motion Sickness (MS) Animal Model

Mice in the experimental group were subjected to 2 h of dual‐axis rotation in complete darkness using a custom‐built rotator combined with a self‐designed apparatus [11]. The rotator system generated compound rotational motion via two vertically oriented turntables with diameters of 80 and 30 cm, respectively. The central axes of the two turntables were separated by a distance of 24 cm. The large turntable rotated clockwise for 15 s and then counterclockwise for 15 s alternately, with a peak angular velocity of 150°/s. The small turntable, which was fixed on the large turntable, rotated continuously counterclockwise at a constant velocity of 450°/s. The directional alternation of the large turntable resulted in an angular acceleration and deceleration of 90°/s^2^. Mice in the control group were placed in the same tubes adjacent to the rotation apparatus for 2 h and received sham stimulation synchronized with the rotation group.

### 2.4 Kaolin Intake

Pica behavior was used to evaluate gastrointestinal discomfort in a mouse model of MS [12]. A non‐nutritive mixture of kaolin and acacia (Sigma‐Aldrich, St. Louis, MI, USA) was prepared by blending pharmaceutical‐grade kaolin with 1% (*w*/*w*) acacia in distilled water to form a thick paste, and then shaped into column‐shaped pellets similar to regular food before drying.

Mice in the experimental group were placed in restraining cages and rotated for 2 h per day for 4 consecutive days. The daily amounts of supplied and residual kaolin were weighed and subtracted, and 4‐day kaolin consumption (measured to the nearest 0.01 g) was calculated for each mouse immediately after treatment.

### 2.5 Motion Sickness (MS) Index Score

Immediately after the final MS stimulation, mice were placed on the ground, and the MS index of the mice was recorded. The method for calculating the MS index score is shown in Table 1; the final score is the sum of the individual scores.

**TABLE 1:** Motion sickness (MS) index score.

MS symptoms	Scores
Fecal granules	None: 0; 1 point per fecal granule
Urination	None: 0; urination: 1.2
Piloerection	None: 0; light: 0.6; severe: 1.2
Tremor	None: 0; tremor: 1.2

Abbreviation: MS, motion sickness.

### 2.6 Open Field Test (OFT)

OFT was performed to assess MS‐related behaviors in mice (Sans Biotechnology Co. Ltd., Jiangsu, China) [13]. Briefly, each mouse was gently placed in the center of the testing arena and allowed to explore freely for 10 min. The “central distance (%)” was calculated as the ratio of central distance to the total distance traveled.

### 2.7 Morris Water Maze

Behavioral training was conducted in accordance with standard routines. The latency to reach the platform was recorded using commercial behavioral analysis software (Mobile Datum Co. Ltd., Shanghai, China). Following the training phase, the test procedure was initiated. Baseline performance was first measured, after which mice received rotational stimulation for 2 h per day over 4 consecutive days, and final behavioral parameters were subsequently assessed. Four indices were analyzed to evaluate MS‐related spatial behaviors: platform crossing times, effective distance, effective time, and effective distance (%). The effective quadrant was defined as the quadrant in which the platform was located. Effective distance and effective time were defined as the distance traveled and the time spent in the effective quadrant, respectively. The “effective distance (%)” was calculated as the ratio of effective distance to the total distance traveled, which normalizes for inter‐individual differences in swimming ability.

### 2.8 CatWalk Gait Analysis

Each mouse was placed at the open end of an enclosed glass platform and allowed to traverse the glass floor voluntarily. During this period, a high‐speed camera mounted beneath the apparatus captured footprint images of each paw, and the corresponding data were transmitted to specialized gait analysis software (CatWalk XT, Version 10.7, Noldus, Wageningen, the Netherlands) for subsequent processing. Six gait parameters were analyzed in the present study: RH stand (the duration of right hind paw contact with the glass floor), RH stand index (an index describing the speed at which the paw leaves the glass floor; faster running yields more negative values), RH mean intensity (the mean pressure exerted by the right hind paw), regularity index (the percentage of step sequences conforming to regular footfall patterns, reflecting interlimb coordination), print position (the distance between the ipsilateral fore‐ and hind paws, reflecting stride compensation), and base of support (the distance between the hind paws, reflecting postural stability).

### 2.9 Tracer and Virus Injection

The stereotaxic injection procedures were performed as previously described [14, 15]. All viruses used in the present study were purchased from BrainVTA Co. Ltd. (Wuhan, China), Brain Case Bioscience Co. Ltd. (Shenzhen, China), and Taitool Bioscience Co. Ltd. (Shanghai, China). Detailed injection parameters are provided below.

For neural tracing, red Retrobeads (Lumaflor Inc., Miramar, USA) were injected into the POR of C57 mice, and scAAV2/1‐hSyn‐tdTomato‐pA (S1165‐1, 1.15 × 10^13^) was injected into the CLA of C57 mice. All injections were performed in accordance with the mouse brain atlas established by Paxinos and Watson. The stereotaxic coordinates for the POR were as follows: −4.60 mm anterior to the bregma, 3.75 mm lateral to the midline, and 3.10 mm ventral to the skull surface. The coordinates of the CLA were as follows: 1.10 mm anterior to the bregma, 2.70 mm lateral to the midline, and 3.85 mm ventral to the skull surface.

For fluorescence micro‐optical sectioning tomography (fMOST), AAV9‐CSSP‐YFP‐2E4 (BC‐SL004, 5.0 × 10^13^) was injected into the CLA of *VGAT‐Cre* or *VGluT2‐Cre* mice.

For optogenetic stimulation, AAV2/9‐hEF1a‐DIO‐hChR2(H134R)‐mCherry‐WPRE‐pA (S0170‐9, 1.83 × 10^13^) was injected into the CLA of *VGAT‐Cre* or *VGluT2‐Cre* mice.

For chemogenetic manipulation, AAV2/9‐EF1a‐DIO‐hM3D(Gq)‐mCherry‐WPREs (PT‐0042, 2.00 × 10^12^), AAV2/9‐EF1a‐DIO‐hM4D(Gi)‐mCherry‐WPREs (PT‐0043, 2.00 × 10^12^), or AAV2/9‐EF1a‐DIO‐mCherry‐WPRE‐hGH pA (PT‐0013, 2.00 × 10^12^) was injected into the CLA, and AAV2/Retro‐VGluT2‐SV40 NLS‐Cre‐hGH polyA (BC‐1114, 2.00 × 10^12^) was injected into the POR of C57 mice. AAV2/9‐GAD67‐DIO‐hM3D(Gq)‐mCherry‐WPREs (PT‐3542, 2.00 × 10^12^), AAV2/9‐GAD67‐DIO‐hM4D(Gi)‐mCherry‐WPRE‐hGH polyA (PT‐0608, 2.00 × 10^12^), or AAV2/9‐GAD67‐DIO‐mCherry‐WPRE‐hGH pA (PT‐1964, 2.00 × 10^12^) was injected into the CLA, and AAV2/Retro‐hSyn‐Cre‐WPRE‐pA (S0278‐2R, 1.75 × 10^13^) was injected into the POR of C57 mice.

For fiber photometry recordings, AAV2/9‐EF1a‐DIO‐GCaMP6s‐EGFP (BC‐0086, 2.00 × 10^12^) was injected into the CLA, AAV2/Retro‐mVGLUT2‐Cre (S1901‐2R‐H5, 2.07 × 10^13^) or AAV2/Retro‐mVGAT1‐Cre (BC‐1723, 5.00 × 10^12^) into the POR of C57 mice. After viral injection, an optical fiber (200 μm outer diameters, 0.37 numerical aperture, Fiblaser, Shanghai, China) was implanted above the CLA.

### 2.10 Fluorescence Micro‐Optical Sectioning Tomography (fMOST)

Whole‐brain imaging of sparsely labeled CLA‐POR neurons was performed using fMOST. Brains with viral injection of AAV9‐CSSP‐YFP‐2E4 were embedded in London Resin (LR)‐White resin and imaged with an fMOST system (Wuhan OE Bio Co. Ltd., Wuhan, China) equipped with a 473‐nm laser, a 40× water‐immersion objective, and a Time Delay and Integration (TDI)‐charge‐coupled device (CCD) detector. Serial coronal sections (2‐μm thickness) were acquired at a voxel resolution of 0.32 × 0.32 × 1.00 μm^3^. Three‐dimensional reconstruction and visualization of single neurons were conducted using Amira software (Thermo Fisher Scientific, Waltham, USA) and Fast Neurite Tracer (State Key Lab of Neurosci, Shanghai Insts for Biol Sci, CAS, Shanghai, China).

### 2.11 Immunohistochemical Staining

Immunofluorescence staining was conducted in accordance with our previously established protocol [16, 17]. Brain sections from the medial vestibular nucleus, parvicellular part (MVePC), CLA, and POR regions were immunostained with a primary antibody, mouse anti‐FOSb (1:3000, ab11959, Abcam, MA, USA). The secondary antibody was Alexa Fluor 488 anti‐mouse (1:400, A21202, Invitrogen, Thermo Fisher, CA, USA).

In order to confirm the projection range, we used latexin (LXN), a CLA marker [18]. Brain slices in the CLA were incubated with a primary antibody, rabbit anti‐LXN (1:200, Cat No. 13056‐1‐AP, Proteintech, Wuhan, China). The secondary antibody was Alexa Fluor 488 anti‐rabbit (1:400, A21206, Invitrogen, Thermo Fisher, CA, USA).

Three days after beads injection into the POR, or 24 days after viral injection of scAAV2/1‐hSyn‐tdTomato‐pA into the CLA, mice were subjected to rotational stimulation. After the last rotation, mice were sacrificed. The sections were incubated with primary antibodies, mouse anti‐FOSb, rabbit anti‐calcium/calmodulin‐dependent protein kinase II (CaMKII, 1:200, ab216503, Abcam, MA, USA), or rabbit anti‐glutamic acid decarboxylase 67 (GAD67, 1:200, ab213508, Abcam, Cambridge, MA, USA). The secondary antibodies were Alexa Fluor 647 anti‐mouse (1:400, A31571, Invitrogen, Thermo Fisher, CA, USA) and Alexa Fluor 488 anti‐rabbit. Imaging was conducted using a confocal microscope (FV3000, Olympus, Tokyo, Japan) or a slide scanner (Slideview, VS200, Olympus, Tokyo, Japan).

After the virus was expressed for 4 weeks and all behavioral experiments were finished, the mice subjected to chemogenetic activation of the CLA^VGluT2^–POR pathway or chemogenetic inhibition of the CLA^GAD67^–POR pathway were subjected to rotational stimulation after the injection of CNO. After the last rotation, mice were sacrificed. The sections were incubated with the primary antibody, mouse anti‐FOSb. The secondary antibody was Alexa Fluor 488 anti‐mouse. Imaging was conducted using a confocal microscope (FV3000, Olympus, Tokyo, Japan) or a slide scanner (Slideview, VS200, Olympus, Tokyo, Japan).

### 2.12 Fluorescence In Situ Hybridization (FISH)

Fluorescence in situ hybridization (FISH) was performed according to previously reported protocols [19]. VGluT1 or VGluT2 riboprobes (YSY Biotech Co. Ltd., Nanjing, China) were added to the prehybridization solution. Primary antibodies were sheep anti‐digoxigenin (DIG)‐peroxidase (POD) Fab fragments (1:100, 11207733910, Roche, Basel, Switzerland), rabbit anti‐red fluorescent protein (RFP, 1:200, ab124754, Abcam, Cambridge, UK), and mouse anti‐FOSb. Secondary antibodies contained Alexa Fluor 594 anti‐rabbit and Alexa Fluor 647 anti‐mouse. Avidin coupled with fluorescein isothiocyanate (FITC) was also applied at the same dilution (1:400, 21221, Invitrogen, Thermo Fisher, CA, USA).

### 2.13 Whole‐Cell Patch‐Clamp Recordings

All experimental protocols adopted in the present study were performed in accordance with previously published methodologies [16]. Coronal brain slices with a thickness of 300 μm containing the POR were prepared using a vibrating microtome (Leica VT 1200s, Nussloch, Germany) at a constant temperature of 4°C. Subsequently, the obtained brain slices were placed in a submerged recovery chamber at room temperature. The chamber was filled with oxygenated artificial cerebrospinal fluid (ACSF) saturated with 95% O_2_ and 5% CO_2_. The ACSF solution contained the following components at corresponding millimolar concentrations: 124 NaCl, 25 NaHCO_3_, 2.5 KCl, 1 NaH_2_PO_4_, 2 CaCl_2_, 1 MgSO_4_, and 10 glucose. Whole‐cell electrophysiological recordings were conducted under voltage‐clamp configuration with an Axon 700B amplifier (Molecular Devices, San Jose, CA, USA). Data acquisition and offline analysis were completed using Clampex and Clampfit 10.2 software (Molecular Devices), respectively. For optogenetic stimulation, ChR2‐positive axon terminals derived from the CLA within the POR region were activated by 470‐nm blue light pulses. The optical stimulation was delivered through a microscope‐coupled LED and optical fiber system, with light power set at 1 to 3 mW and pulse duration fixed at 5 ms. During recordings, optically evoked excitatory postsynaptic currents (oEPSCs) were collected from *VGluT2‐Cre* mice, while optically evoked inhibitory postsynaptic currents (oIPSCs) were recorded from *VGAT‐Cre* mice. Cells were voltage‐clamped at −70 mV for EPSC recordings and at 0 mV for IPSC recordings. For oEPSCs and oIPSCs recordings, the pipette solution contained (in mM): 112 Cs‐gluconate, 5 TEA‐Cl, 3.7 NaCl, 0.2 EGTA, 10 HEPES, 2 MgATP, 0.3 Na_3_GTP, and 5 QX‐314 (pH 7.2 with CsOH; ~290 mOsm). For monosynaptic investigations, 6‐cyano‐7‐nitroquinoxaline‐2,3‐dione disodium salt hydrate (CNQX, 20 μM), d‐2‐amino‐5‐phosphonopentanoic acid (D‐AP5, 50 μM), and 2‐(3‐carboxypropyl)‐3‐amino‐6‐(4 methoxyphenyl)‐pyridazinium bromide (Gabazine, 10 μM) were added to the ACSF. Target neurons receiving synaptic contacts from mCherry‐labeled axon terminals in the POR were visually identified using an Olympus BX51WI fluorescence microscope. A final concentration of 0.2% biocytin (Sigma‐Aldrich, St. Louis, MI, USA) was added to the intracellular recording solution to label and trace the recorded neurons. Optical stimulation signals at 470 nm were generated by a pE‐300ultra illumination system (CoolLED, Andover, UK) mounted on the microscope. Blue light stimuli were delivered through a 40× objective lens positioned above the visual field of patched neurons.

### 2.14 Fiber Photometry

Fluorescence signals (excitation: 470/410 nm) were acquired with a RWD photometry system (RWD Life Science Co. Ltd., Shenzhen, China). Following 4 consecutive days of MS stimuli (2 h per day), mice were transferred to the testing room, and a 10‐min baseline recording was performed immediately (within 10 min) after the cessation of the final rotation session. Signals were sampled at 50 Hz with 10%–20% light intensity. Δ*F*/*F* was computed as (*F* − *F*_0_)/*F*_0_, where *F*_0_ represents the baseline mean fluorescence. Animals exhibiting incorrect viral expression or optical fiber misplacement were excluded from subsequent analyses.

### 2.15 Statistical Analysis

Experiments were carried out in a randomized, blinded manner with parallel control groups. Data are presented as mean ± SEM. Statistical analyses were performed with GraphPad Prism 9.0 software (GraphPad Software Inc., San Diego, CA, USA). For single behavioral comparisons between two groups (control vs. MS mice, mCherry vs. hM3Dq, mCherry vs. hM4Di, Saline vs. CNO), statistical significance was determined using an unpaired two‐tailed Student's *t*‐test (Figures 1, 2, 3, 5, 6, S2, S3, S7–S12, S14 and S15). For FOS quantification across multiple brain regions (MVePC, CLA, POR) at multiple time points (Day 0, Day 1, Day 3, Day 5, Day 7), two‐way mixed‐effects ANOVA with Bonferroni's post hoc test was applied (Figure S4). All *p*‐values were based on two‐tailed tests. For all analyses, *p* < 0.05 was considered statistically significant.

## 3 Results

### 3.1 MS Impaired Spatial Orientation and Induced Gait Abnormalities in Male Mice

To induce MS in mice, we employed a double‐axis rotation apparatus previously established in our laboratory (Patent number: CN201375509Y) [11]. Male mice were subjected to dual‐axis rotation for 4 consecutive days. Behavioral tests were performed immediately after the last round of rotation (Figures 1A and S1). Kaolin intake, MS index, and OFT results showed that MS mice exhibited a non‐significant increasing trend in pica behavior, a significantly increased MS index, and a marked reduction in spontaneous locomotor activity, confirming successful establishment of the MS model (Figures 1B–D and S3B). To further investigate the impact of MS on spatial orientation, we performed the Morris water maze test. MS mice exhibited significant reductions in platform crossing times, effective distance, effective time, and effective distance percentage, indicating marked spatial orientation deficits (Figure 1E,F).

Previous rodent MS models have been predominantly established in rats, with kaolin intake (pica behavior) serving as the standard indicator [12, 20]. However, relying on a single behavioral measure has inherent limitations. In this study, we adopted a mouse model, given the wider applicability of mice in translational research, although kaolin intake is relatively unstable [21]. We therefore integrated the CatWalk gait analysis system to assess motor function. Six parameters were selected for comprehensive gait profiling analysis: RH stand, RH stand index, RH mean intensity, Regularity index, Print Position (right paws), and Base of Support (hind paws). These parameters capture gait across speed, pressure, and coordination dimensions. All six parameters were significantly reduced in MS mice, indicating hurried gait, reduced paw pressure, disordered step sequences, shortened stride length, and compromised postural support (Figure 1G,H). Collectively, these pronounced gait abnormalities demonstrated that CatWalk analysis detects MS‐related motor impairments. Notably, the total distance traveled during the Morris water maze task remained unchanged in MS mice (Figure S3A), which contrasts with the results observed in OFT (Figure 1D). We speculate that the gait abnormalities induced by MS may arise from impaired postural balance rather than primary structural damage to locomotor tissues. This alteration likely reflects a compensatory protective mechanism rather than a genuine motor deficit. Swimming in the water maze primarily relies on propulsive forces generated by limb paddling, and the buoyancy provided by water reduces the demand for postural control. Consequently, swimming is less dependent on posture than terrestrial locomotion [22].

In contrast, female mice exhibited largely intact locomotor, spatial, and gait performance under identical stimulation (Figures S2 and S3C), with the exception of a trend toward an increased MS index that did not reach statistical significance (Figure S3D). These results indicate that female mice are relatively resistant to the behavioral manifestations of MS under this stimulation paradigm. Therefore, all subsequent experiments were conducted using male mice.

### 3.2 MS Induced Differential FOS Expression in the MVePC, CLA, and POR

To further assess the reliability and feasibility of the model, we performed immunofluorescence staining combined with FISH to investigate the underlying mechanisms. Whole‐brain FOS screening revealed increased FOS expression in the medial vestibular nucleus (MVe), further validating the model (Figure S4A–D). In addition, increased numbers of FOS^+^ neurons were observed in several diencephalic regions, including the lateral hypothalamic area (LHA) and the zona incerta (ZI) (Figure S4A,B). Given that the vestibular system sends direct projections to the thalamus [23], the observed activation in these diencephalic nuclei may reflect secondary engagement via thalamo‐diencephalic circuits [24, 25].

Notably, we observed an overall trend of decreased FOS^+^ neurons across brain regions, with the most pronounced changes occurring in the CLA, whereas the POR exhibited only a modest change, suggesting that these two brain regions may be involved in the central regulation of MS (Figure S4A,B). Given the involvement of the POR in spatial orientation processing [9, 10], subsequent experiments were primarily focused on the CLA and POR. Quantitative immunofluorescence staining revealed that, compared with the control group, the number of FOS^+^ neurons in the MVePC was significantly increased on the day of induction and on the first day after MS, whereas CLA and POR FOS^+^ counts were significantly decreased only on the day of model induction, and they all gradually recovered within 7 days (Figure S4C–H). Collectively, these results suggest that MS entails enhanced MVe activity alongside functional suppression of the CLA and POR, suggesting a hypoactive or functionally impaired circuit state.

### 3.3 Opposing Changes in Glutamatergic and GABAergic CLA–POR Neuron Activity in MS

To determine whether the observed FOS suppression reflected relevant circuit dysfunction rather than global hypometabolism, we examined the excitatory‐inhibitory (E/I) composition of CLA–POR projections using combined immunofluorescence staining and FISH on control and MS mice. Following retrograde bead injection into the POR, CLA brain sections revealed extensive colocalization of beads with LXN, confirming that the POR received projections from the CLA (Figure 2A,B). Compared with the control group, the co‐labeling Beads/FOS neurons in MS mice was decreased, indicating reduced excitability of neurons projecting from the CLA to the POR (Figure 2D).

**FIGURE 2:**
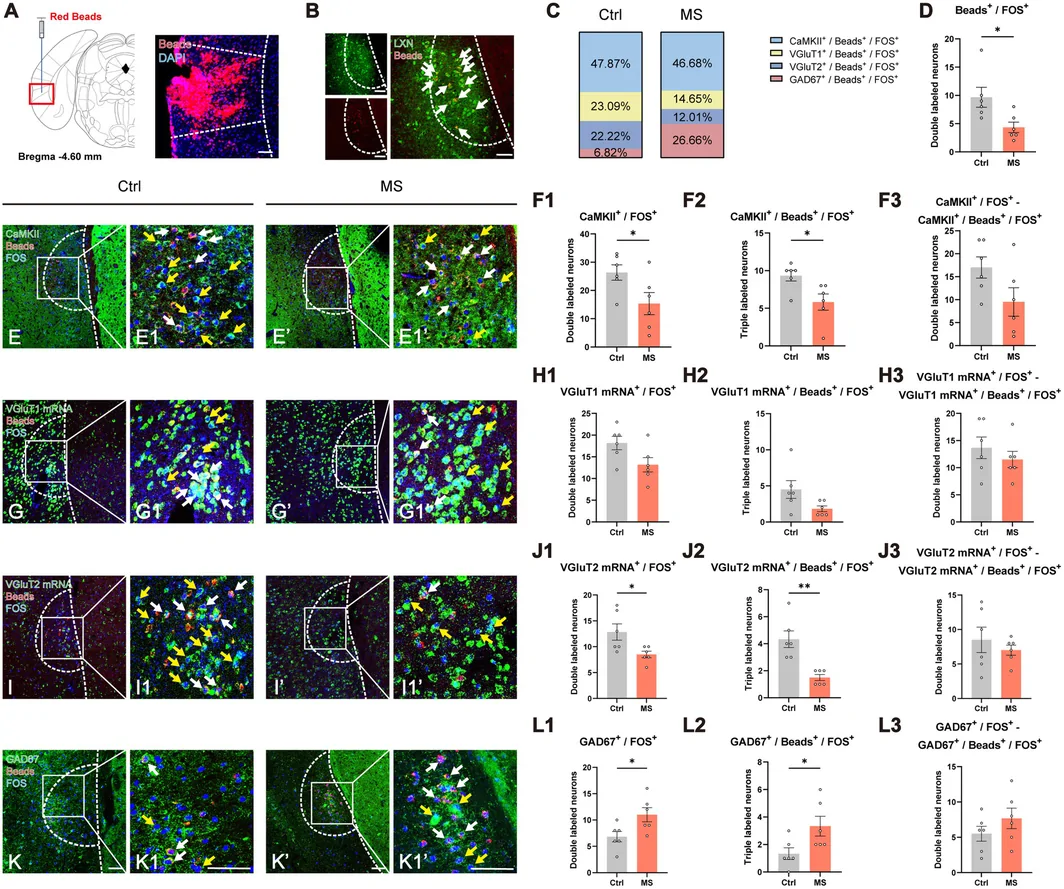
The FOS expression in distinct subtypes of neurons projecting from claustrum (CLA) to postrhinal cortex (POR) in MS mice. (A) Schematic of red Retrobeads injection into the POR, and representative confocal image showing injection site for beads in the POR with DAPI‐counterstained. (B) Confocal images showing the latexin (LXN) immunoreactive (green) and beads^+^ (red) neurons in the CLA. (C) Triple‐labeling probability histogram chart of calcium/calmodulin‐dependent protein kinase II (CaMKII)/Beads/FOS, vesicular glutamate transporter 1 (VGluT1) mRNA/Beads/FOS, VGluT2 mRNA/Beads/FOS, and glutamic acid decarboxylase 67 (GAD67)/Beads/FOS, the percentage is expressed as the proportion of specific trichromatic neurons to the total trichromatic neurons. (D) The double labeled neurons of Beads/FOS in the CLA (*n* = 6 sections/3 mice per group). (E) Confocal images showing the CaMKII immunoreactive (green), beads^+^ (red), and FOS immunoreactive (blue) neurons in the CLA from the indicated groups. (F) The double labeled neurons of CaMKII/FOS, triple labeled neurons of CaMKII/Beads/FOS, and double labeled neurons of CaMKII/FOS (triple labeled neurons are excluded) in the CLA (*n* = 6 sections/3 mice per group). (G) Confocal fluorescence in situ hybridization (FISH) combined with immunofluorescence staining images showing VGluT1 mRNA^+^ (green), beads^+^ (red), and FOS^+^ neurons (blue) in the CLA. (H) The double labeled neurons of VGluT1 mRNA/FOS, triple labeled neurons of VGluT1 mRNA/Beads/FOS, and double labeled neurons of VGluT1 mRNA/FOS (triple labeled neurons are excluded) in the CLA (*n* = 6 sections/3 mice per group). (I) Confocal FISH combined with immunofluorescence staining images showing VGluT2 mRNA^+^ (green), beads^+^ (red), and FOS^+^ neurons (blue) in the CLA. (J) The double labeled neurons of VGluT2 mRNA/FOS, triple labeled neurons of VGluT2 mRNA/Beads/FOS, and double labeled neurons of VGluT2 mRNA/FOS (triple labeled neurons are excluded) in the CLA (*n* = 6 sections/3 mice per group). (K) Confocal images showing the GAD67 immunoreactive (green), beads^+^ (red), and FOS immunoreactive (blue) neurons in the CLA from the indicated groups. (L) The double labeled neurons of GAD67/FOS, triple labeled neurons of GAD67/Beads/FOS, and double labeled neurons of GAD67/FOS (triple labeled neurons are excluded) in the CLA (*n* = 6 sections/3 mice per group). Dashed lines delineate brain region boundaries; solid boxes indicate magnified areas. Arrows indicate triple‐labeled (white) or double‐labeled (yellow) neurons. Scale bar, 40 μm. Data are mean ± SEM; statistical analyses were conducted using unpaired *t*‐test; *p < 0.05, **p < 0.01. Statistical details are provided in Table S1. CaMKII, calcium/calmodulin‐dependent protein kinase II; Ctrl, control; GAD67, glutamic acid decarboxylase 67; LXN, latexin; MS, motion sickness; VGluT1, vesicular glutamate transporter 1.

Triple‐labeling analysis revealed that, compared with the control group, MS mice had fewer CLA–POR projection neurons positive for CaMKII/Beads/FOS and VGluT2 mRNA/Beads/FOS (Figure 2C,E,F,I,J), alongside an increased proportion of GAD67/Beads/FOS projection neurons (Figure 2C,K,L). These data indicate that MS reduces glutamatergic and increases GABAergic drive from the CLA to the POR. Consistent with this shift, double labeling for CaMKII/FOS and VGluT2 mRNA/FOS was decreased (Figure 2E,F,I,J), whereas GAD67/FOS double labeling was increased (Figure 2K,L). Among non‐POR‐projecting CLA neurons, excitatory markers showed a decreasing trend and inhibitory markers an increasing trend (Figure 2E–L). This finding suggests that the reduction in FOS^+^ neurons in the CLA of MS mice reflects a dual mechanism involving both extrinsic afferent suppression and intrinsic microcircuit reorganization. In contrast, the colocalization levels of VGluT1 mRNA/Beads/FOS and VGluT1 mRNA/FOS showed no significant changes, suggesting that VGluT1^+^ CLA neurons are not engaged in MS (Figure 2G,H). We also confirmed that VGluT2^+^ and GAD67^+^ neurons within the CLA → POR projection pathway are mutually exclusive populations (Figure S5). Among Beads^+^ neurons, 17.06% ± 2.38% were VGluT2^+^, 9.34% ± 0.96% were GAD67^+^, and only 3.31% ± 0.59% co‐expressed both markers (Figure S5B), indicating that the two projection populations are largely mutually exclusive.

In the POR, anterograde transsynaptic labeling with scAAV2/1‐hSyn‐tdTomato from the CLA showed decreased tdTomato/FOS, CaMKII/tdTomato/FOS, and VGluT1 mRNA/tdTomato/FOS triple labeling in MS mice, whereas VGluT2 mRNA/tdTomato/FOS and GAD67/tdTomato/FOS levels showed no significant change (Figures 3 and S13A). This indicated a decrease in the activity of POR neurons receiving CLA innervation in MS mice. Based on these observations, we focused subsequent functional analyses on the excitatory and inhibitory CLA neuron populations. First, to verify that CLA can project long‐range to the POR, we performed whole‐cell patch‐clamp recordings. The results showed that oEPSCs could be evoked in the POR of *VGluT2‐Cre* mice injected with AAV2/9‐hEF1a‐DIO‐hChR2(H134R)‐mCherry in the CLA (Figure S6A–E), whereas oIPSCs were evoked in *VGAT‐Cre* mice (Figure S6F–J). Specifically, the oIPSCs were not affected by glutamatergic receptor antagonists and were completely blocked by the GABA_A_ receptor antagonist gabazine (Figure S6J). This provided clear evidence of long‐range projections.

**FIGURE 3:**
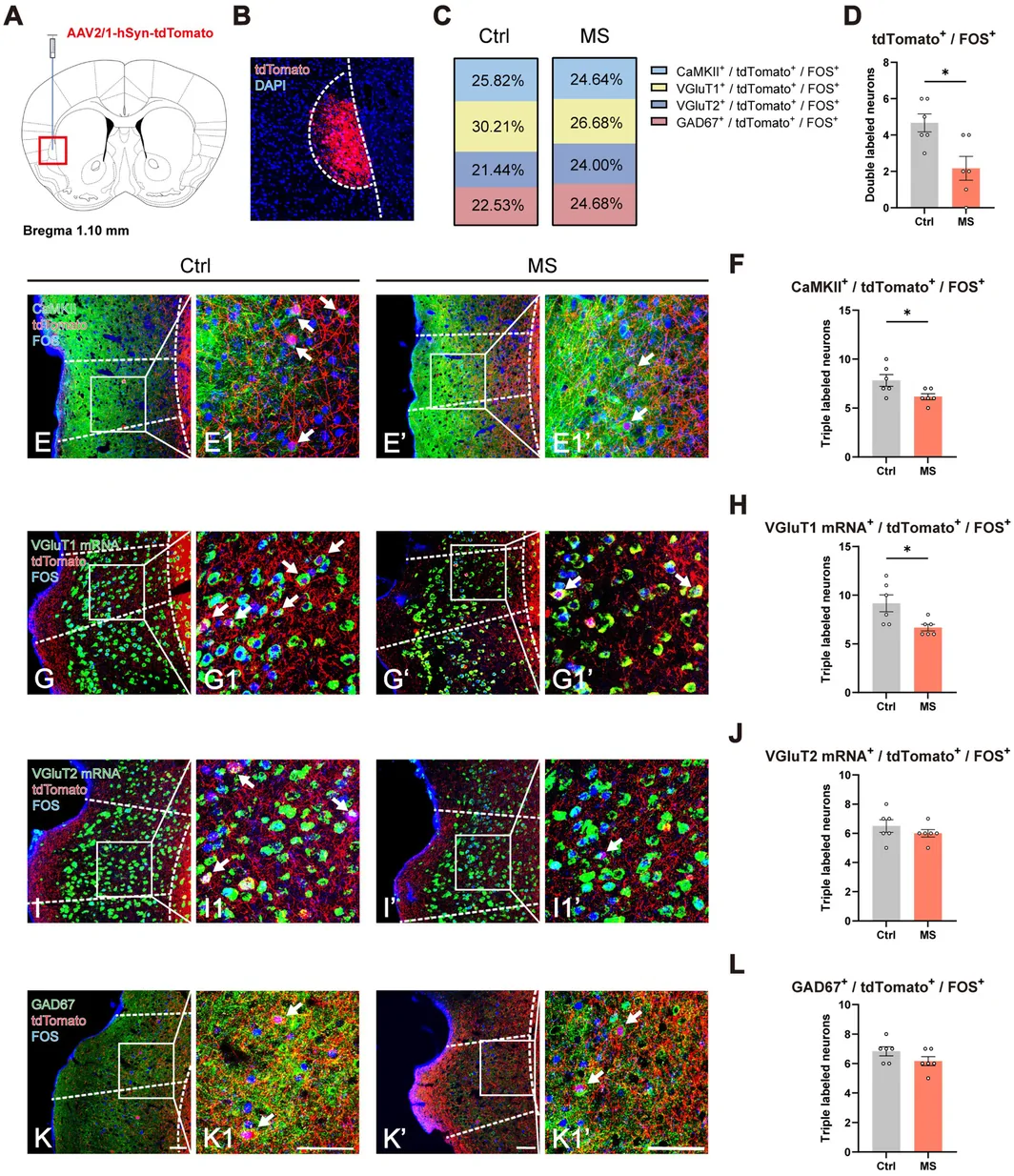
FOS expression in distinct neuronal subtypes of POR innervated by CLA in MS mice. (A) Schematic of viral injection of AAV2/1‐hSyn‐tdTomato into the CLA. (B) Representative confocal image showing injection site for tdTomato expression in the CLA with DAPI‐counterstained. (C) Triple‐labeling probability histogram chart of CaMKII/tdTomato/FOS, VGluT1 mRNA/tdTomato/FOS, VGluT2 mRNA/tdTomato/FOS, and GAD67/tdTomato/FOS, the percentage is expressed as the proportion of specific trichromatic neurons to the total trichromatic neurons. (D) The double labeled neurons of tdTomato/FOS in the POR (*n* = 6 sections/3 mice per group). (E) Confocal images showing the CaMKII immunoreactive (green), tdTomato^+^ (red), and FOS immunoreactive (blue) neurons in the POR from the indicated groups. (F) The triple labeled neurons of CaMKII/tdTomato/FOS in the POR (*n* = 6 sections/3 mice per group). (G) Confocal FISH combined with immunofluorescence staining images showing VGluT1 mRNA^+^ (green), tdTomato^+^ (red), and FOS^+^ neurons (blue) in the POR. (H) The triple labeled neurons of VGluT1 mRNA/tdTomato/FOS in the POR (*n* = 6 sections/3 mice per group). (I) Confocal FISH combined with immunofluorescence staining images showing VGluT2 mRNA^+^ (green), tdTomato^+^ (red), and FOS^+^ neurons (blue) in the POR. (J) The triple labeled neurons of VGluT2 mRNA/tdTomato/FOS in the POR (*n* = 6 sections/3 mice per group). (K) Confocal images showing the GAD67 immunoreactive (green), tdTomato^+^ (red) and FOS immunoreactive (blue) neurons in the POR from the indicated groups. (L) The triple labeled neurons of GAD67/tdTomato/FOS in the POR (*n* = 6 sections/3 mice per group). White dashed line, the extent of the brain region. White solid lines, the magnified regions. White arrows, triple labeled neurons. Scale bar, 40 μm. Data are mean ± SEM; statistical analyses were conducted using unpaired *t*‐test; *p < 0.05. Statistical details are provided in Table S1. CaMKII, calcium/calmodulin‐dependent protein kinase II. Ctrl, control; GAD67, glutamic acid decarboxylase 67; MS, motion sickness; VGluT1, vesicular glutamate transporter 1.

Next, to resolve the projection architecture of these populations, we injected the sparse labeling virus AAV9‐CSSP‐YFP‐2E4 into the CLA of *VGAT‐Cre* and *VGluT2‐Cre* mice, followed by whole‐brain fMOST. After quality control in one randomly selected mouse per cohort, the remaining brains were dehydrated, embedded, and imaged at single‐cell resolution (Figure 4A–C). Three‐dimensional reconstruction revealed two distinct projection patterns from the CLA to the POR. Specifically, GABAergic neurons exhibited a broad and diffuse projection pattern, characterized solely by ipsilateral projections (Figure 4D). In contrast, glutamatergic neurons exhibited a long‐range and precise projection pattern, targeting predominantly ipsilateral regions while also sending a small number of contralateral projections (Figure 4E). Quantitative analysis revealed that CLA neurons projecting to the POR exhibited dense innervation in the visual areas (VIS), the retrosplenial area (RSP), and the entorhinal cortex (ENT). This finding suggests that the CLA exhibits relatively high projection density to core spatial cognition regions, further underscoring the key role of the CLA–POR pathway in regulating spatial orientation function during MS (Figure 4F). Additionally, CLA axons that extended long projections to the neocortex and hippocampus correspond to the integration of core functions such as memory, cognition, sensation, and movement [26, 27, 28] (Figure 4G). These divergent projection patterns suggest that different subtypes of CLA–POR neurons may cooperate to regulate local POR circuits, cortical integration, and thalamic ascending transmission, thereby participating in multisensory integration and contributing to the processing of sensory conflict and the modulation of MS symptoms.

**FIGURE 4:**
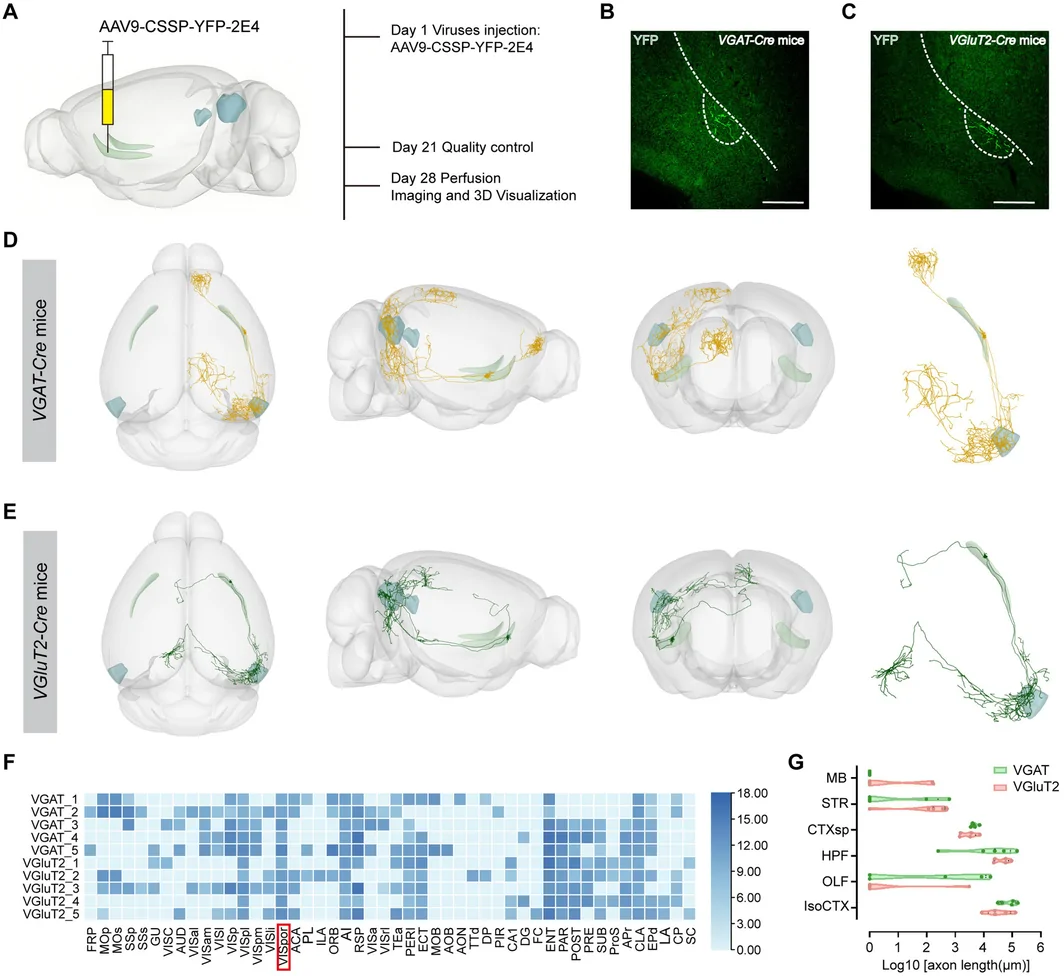
Three‐dimensional reconstruction of the soma, dendrites, and axons of a single CLA‐POR neuron. (A) Schematic of viral injection of AAV9‐CSSP‐YFP‐2E4 and experimental timeline. (B) Representative confocal image showing injection site in the CLA of *vesicular GABA transporter (VGAT)‐Cre* mice. White dashed line, the extent of the brain region. Scale bar, 200 μm. (C) Representative confocal image showing injection site in the CLA of *VGluT2‐Cre* mice. White dashed line, the extent of the brain region. Scale bar, 200 μm. (D, E) Horizontal, sagittal, coronal, and amplificatory views of CLA neurons projecting to the POR. The gray outline represents the mouse brain. The green region indicates the CLA. The blue region indicates the POR. Yellow and green lines represent projecting axonal fibers. (F) Heatmap for the axonal projection intensity of CLA^VGAT^‐POR and CLA^VGluT2^‐POR projection neurons to specific brain regions throughout the brain (*n* = 5 mice per group). (G) The axonal projection intensity of CLA^VGAT^‐POR and CLA^VGluT2^‐POR projection neurons to MB, STR, CTXsp, HPF, OLF, and IsoCTX. ACA, anterior cingulate area; AI, agranular insular area; AOB, accessory olfactory bulb; AON, anterior olfactory nucleus; APr, area prostriata; AUD, auditory areas; CLA, claustrum; CP, caudoputamen; CTXsp, cortical subplate; DG, dentate gyrus; DP, dorsal peduncular cortex; ECT, ectorhinal area; ENT, entorhinal area; EPd, endopiriform nucleus, dorsal part; FC, fasciola cinerea; FRP, frontal pole, cerebral cortex; GU, gustatory areas; HPF, hippocampal formation; ILA, infralimbic area; IsoCTX, isocortex; LA, lateral amygdalar nucleus; MB, midbrain; MOB, main olfactory bulb; MOp, primary motor area; MOs, secondary motor area; OLF, olfactory areas; ORB, orbital area; PAR, parasubiculum; PERI, perirhinal area; PIR, piriform area; PL, prelimbic area; POST, postsubiculum; PRE, presubiculum; ProS, prosubiculum; RSP, retrosplenial area; SC, superior colliculus; SSp, primary somatosensory area; SSs, supplemental somatosensory area; STR, striatum; SUB, subiculum; TEa, temporal association areas; TTd, taenia tecta, dorsal part; VGAT, vesicular GABA transporter; VGluT2, vesicular glutamate transporter 2; VISa, anterior area; VISal, anterolateral visual area; VISam, anteromedial visual area; VISC, visceral area; VISl, lateral visual area; VISli, laterointermediate area; VISp, primary visual area; VISpl, posterolateral visual area; VISpm, posteromedial visual area; VISpor, postrhinal area; VISrl, rostrolateral visual area.

### 3.4 Activation of the CLA^VGluT2^ –POR Pathway Alleviated MS Symptoms

Given the anatomical diversity of CLA‐POR projection patterns, we hypothesized that distinct cell types might differentially regulate spatial versus motor symptoms. First, we used chemogenetic approaches to specifically manipulate this pathway and assess its effects on MS. MS significantly reduced FOS expression in VGluT2 mRNA^+^ CLA‐POR projection neurons, whereas VGluT1 mRNA^+^ neurons were not engaged (Figure 2G–J). Because the CaMKII promoter mainly labels VGluT1^+^ excitatory populations and a small number of VGluT2^+^ neurons in the CLA [29], a CaMKII‐driven strategy would lack selectivity for the MS‐responsive subpopulation. In MS mice, the changes in triple‐positive neurons of VGluT2 mRNA/Beads/FOS were more pronounced than those of CaMKII/Beads/FOS (Figure 2E,F,I,J). We therefore used a VGluT2‐Cre driver to selectively target the excitatory subpopulation whose activity was actually altered by MS. AAV2/Retro‐VGluT2‐SV40 NLS‐Cre was injected into the bilateral POR of mice, and AAV2/9‐EF1a‐DIO‐hM3D(Gq)‐mCherry or AAV2/9‐EF1a‐DIO‐hM4D(Gi)‐mCherry was injected into the bilateral CLA (Figures 5A,B, S7A,B, S9A,B, and S13B,D,F). Control mice received injections of AAV2/9‐EF1a‐DIO‐mCherry. Behavioral tests revealed that activation of the CLA^VGluT2^–POR pathway restored spontaneous activity and improved spatial cognitive performance in MS mice, as evidenced by increases in platform crossing times, effective distance, effective time, and effective distance percentage in the Morris water maze test, whereas gait parameters showed no significant changes (Figure 5C–H). Activation of CLA^VGluT2^–POR projections increased FOS expression in the POR, demonstrating that manipulation of CLA projection neurons alters downstream POR neuronal activity in a manner consistent with the behavioral rescue (Figure S14A–C). Inhibition of the CLA^VGluT2^–POR pathway did not produce significant alterations in any behavioral test in either MS or C57 mice (Figures S7C–H and S9C–H). Collectively, these findings suggest that the CLA^VGluT2^–POR pathway may regulate spatial orientation ability under pathological conditions without exerting adverse effects in healthy animals. Activation of this pathway alleviates spatial disorientation and restores spontaneous locomotor activity in MS mice, whereas gait parameters remain unaffected.

**FIGURE 5:**
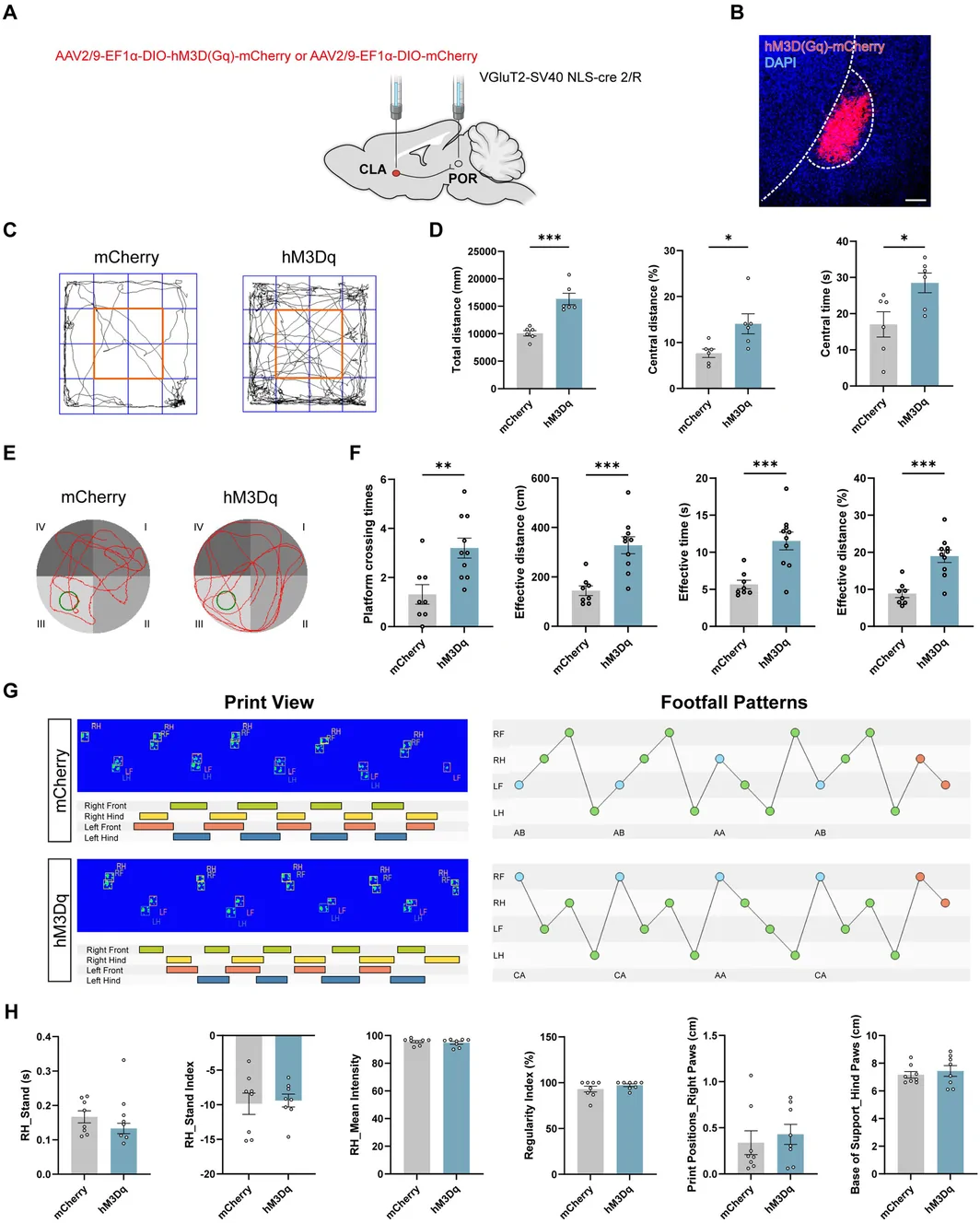
Chemogenetic activation of the CLA^VGluT2^‐POR pathway alleviates MS‐like behaviors. (A) Schematic of viral injection of AAV2/9‐EF1a‐DIO‐hM3D(Gq)‐mCherry or AAV2/9‐EF1a‐DIO‐mCherry into the CLA, and rAAV2/Retro‐VGluT2‐SV40 NLS‐Cre into the POR. (B) Representative confocal image showing injection site for hM3D(Gq) expression in the CLA with DAPI‐counterstained. Scale bar, 100 μm. (C) Representative activity track maps of spontaneous activity from the indicated groups. (D) Total distance, central distance (%), and central time for OFT in mice (*n* = 6 mice per group). (E) Representative activity track maps of Morris water maze from the indicated groups. (F) Platform crossing times, effective distance, effective time, and effective distance (%) for Morris water maze in mice (*n* = 8 mice in mCherry group, *n* = 10 mice in hM3Dq group). (G) The print view and footfall patterns of the step sequence in a mouse that passes through the glass pane. AB, alternate pattern (LF–RH–RF–LH). AA, alternate pattern (RF–RH–LF–LH). CA, cruciate pattern (RF–LF–RH–LH). (H) RH stand, RH stand index, RH mean intensity, Regularity index, Print Positions right paws, and Base of Support hind paws for CatWalk gait analysis in mice (*n* = 8 mice per group). Data are mean ± SEM; statistical analyses were conducted using unpaired *t*‐test; *p < 0.05, **p < 0.01, ***p < 0.001. Statistical details are provided in Table S1. CLA, claustrum; LF, left front; LH, left hind; POR, postrhinal cortex; RF, right front; RH, right hind.

Furthermore, to determine whether the behavioral rescue observed following CNO administration was attributable to the therapeutic effect of the pathway itself rather than potential off‐target effects of the drug, we injected saline into hM3D(Gq)‐injected mice prior to MS induction as a control condition and assessed behavioral changes in MS animals (Figure S11A,B). The results demonstrated that, compared with the saline group, the CNO group exhibited improved spontaneous activity and spatial exploration, whereas gait parameters remained unchanged (Figure S11C–H). This is consistent with our hypothesis.

### 3.5 Inhibition of the CLA^GAD67^ –POR Pathway Alleviated MS Symptoms

To investigate the causal role of the CLA^GAD67^–POR pathway in MS, we performed bidirectional chemogenetic manipulation. AAV2/Retro‐hSyn‐Cre was delivered bilaterally into the POR, while either AAV2/9‐GAD67‐DIO‐hM3D(Gq)‐mCherry or AAV2/9‐EF1a‐GAD67‐DIO‐hM4D(Gi)‐mCherry was injected into the bilateral CLA; control mice received the DIO‐mCherry vector (Figures 6A,B, S8A,B, S10A,B, and S13C,E,G). Behavioral tests revealed that inhibition of the CLA^GAD67^–POR pathway markedly attenuated MS‐related deficits (Figure 6C–H). In the Morris water maze, MS mice showed increased platform crossing times, effective distance, effective time, and effective distance percentage (Figure 6E,F). Concomitantly, there were significant improvements in gait (Figure 6G,H), indicating restored motor coordination and postural stability. Inhibition of CLA^GAD67^–POR projections increased FOS expression in the POR, demonstrating that manipulation of CLA projection neurons alters downstream POR neuronal activity in a manner consistent with the behavioral rescue (Figure S14D–F). By contrast, activation of this pathway produced no significant changes in any behavioral test in either MS or C57 mice (Figures S8C–H and S10C–H). Collectively, these findings suggest that the CLA^GAD67^–POR pathway is necessary for the expression of MS‐related spatial disorientation and gait abnormalities. Selective inhibition of this inhibitory projection alleviates both cognitive and motor symptoms without adverse effects in healthy animals, identifying a circuit‐relevant therapeutic target.

**FIGURE 6:**
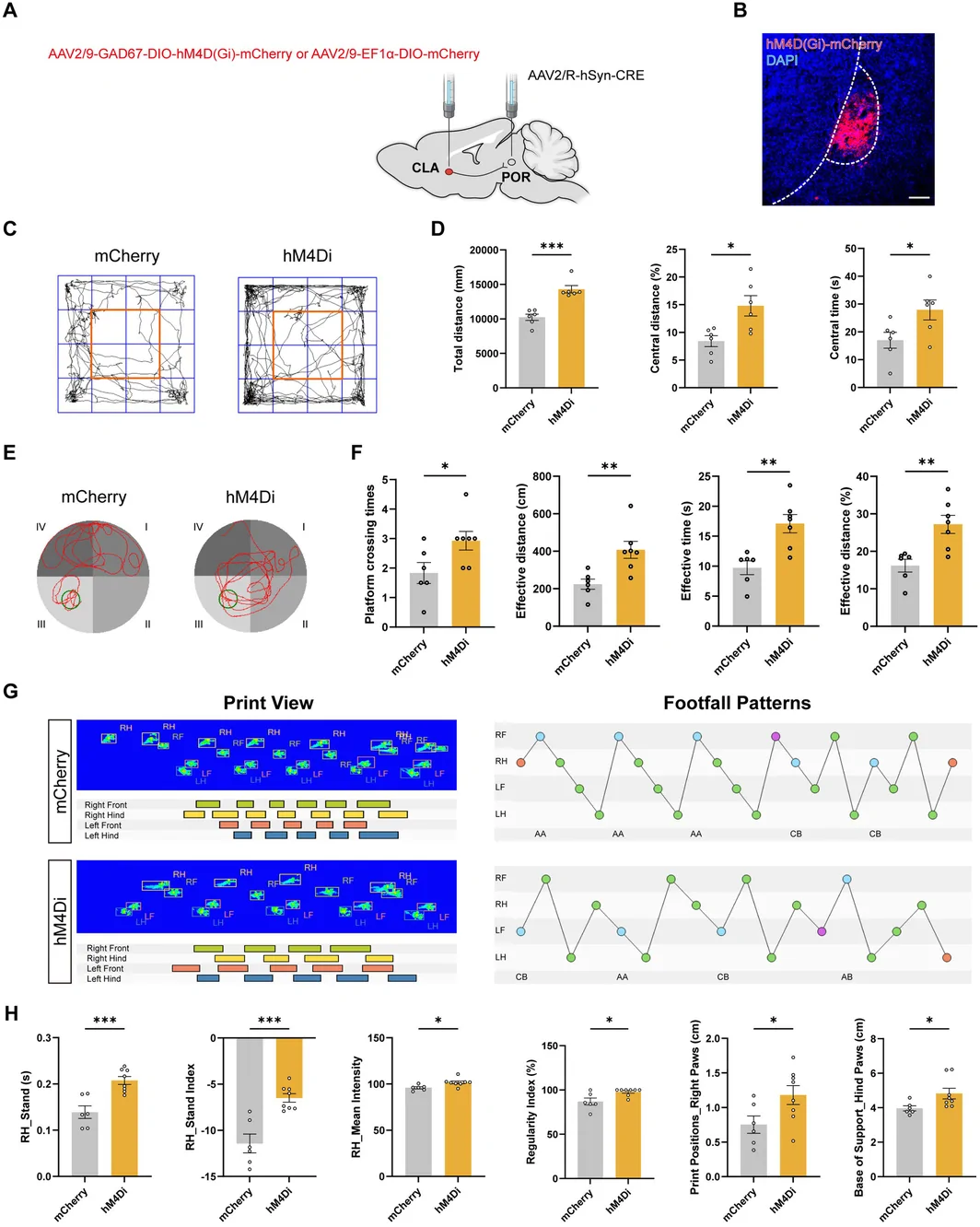
Chemogenetic inhibition of the CLA^GAD67^‐POR pathway alleviates MS‐like behaviors. (A) Schematic of viral injection of AAV2/9‐GAD67‐DIO‐hM4D(Gi)‐mCherry or AAV2/9‐GAD67‐DIO‐mCherry into the CLA, and rAAV2/Retro‐hSyn‐CRE into the POR. (B) Representative confocal image showing injection site for hM4D(Gi) expression in the CLA with DAPI‐counterstained. Scale bar, 100 μm. (C) Representative activity track maps of spontaneous activity from the indicated groups. (D) Total distance, central distance (%), and central time for OFT in mice (*n* = 6 mice per group). (E) Representative activity track maps of Morris water maze from the indicated groups. (F) Platform crossing times, effective distance, effective time, and effective distance (%) for Morris water maze in mice (*n* = 6 mice in mCherry group, *n* = 7 mice in hM4Di group). (G) The print view and footfall patterns of the step sequence in a mouse that passes through the glass pane. AA, alternate pattern (RF–RH–LF–LH). CB, cruciate pattern (LF–RF–LH–RH). AB, alternate pattern (LF–RH–RF–LH). (H) RH stand, RH stand index, RH mean intensity, Regularity index, Print Positions right paws, and Base of Support hind paws for CatWalk gait analysis in mice (*n* = 6 mice in mCherry group, *n* = 8 mice in hM4Di group). Data are mean ± SEM; statistical analyses were conducted using unpaired *t*‐test; *p < 0.05, **p < 0.01, ***p < 0.001. Statistical details are provided in Table S1. CLA, claustrum; LF, left front; LH, left hind; POR, postrhinal cortex; RF, right front; RH, right hind.

Moreover, to determine whether the behavioral rescue observed following CNO administration was attributable to the therapeutic effect of the pathway itself rather than potential off‐target effects of the drug, we injected saline into hM4D(Gi)‐injected mice prior to MS induction as a control condition and assessed behavioral changes in MS animals (Figure S12A,B). The results demonstrated that, compared with the saline group, the CNO group exhibited significant improvements in spontaneous activity, spatial exploration, and gait parameters (Figure S12C–H). These findings confirm our hypothesis.

### 3.6 Fiber Photometry Recordings Confirmed an Excitation/Inhibition (E/I) Imbalance Within the CLA–POR Pathway

Finally, to directly interrogate the E/I imbalance within the CLA–POR pathway at a functional level, we performed fiber photometry calcium imaging to monitor activity changes in CLA projection neurons following MS induction. AAV2/9‐EF1a‐DIO‐GCaMP6s was delivered bilaterally into the CLA, while either AAV2/Retro‐mVGLUT2‐Cre or AAV2/Retro‐mVGAT1‐Cre was injected into the bilateral POR (Figure S15A,B,F,G). As shown in the heatmaps, averaged Δ*F*/*F* traces, and area under the curve (AUC) analyses, the fluorescence signal in the MS group was significantly lower than in the control group of the CLA^VGluT2^–POR pathway (Figure S15C–E), while the fluorescence signal in the MS group was significantly higher than in the control group of the CLA^VGAT^–POR pathway (Figure S15H–J). These complementary functional data provide direct evidence that MS induces an E/I imbalance within the CLA–POR pathway, in agreement with the FOS and chemogenetic findings.

## 4 Discussion

The present study identifies a previously unrecognized central mechanism contributing to MS pathophysiology in mice. By integrating a custom‐built double‐axis rotation apparatus with multi‐dimensional behavioral phenotyping, we demonstrated that the CLA–POR pathway exerts bidirectional regulatory control over MS severity and associated spatial disorientation. Specifically, our findings show that the balance between excitatory and inhibitory projections originating in the CLA within the CLA–POR pathway is an important determinant of MS‐related behavioral deficits. These findings extend our understanding of central mechanisms underlying MS beyond traditional brainstem‐centric models and provide a circuit‐based framework for therapeutic intervention. Collectively, this work establishes the CLA–POR pathway as a viable target for modulating MS severity.

To characterize MS‐related behavioral deficits, we first validated the rotational paradigm through multiple convergent lines of evidence. MS mice exhibited an increase in MS index, significantly reduced locomotor activity in the OFT, impaired spatial memory in the Morris water maze, and pronounced gait abnormalities in CatWalk gait analysis. At the neural level, increased FOS expression in the MVePC confirmed effective vestibular activation, a well‐established physiological indicator of vestibular stimulation. These behavioral and physiological readouts collectively confirm that the dual‐axis rotation paradigm reliably induced MS‐like responses in mice, providing a valid foundation for subsequent circuit‐level analyses. Previous behavioral assessments of MS have primarily focused on pica behavior [12, 30], MS index [31], and OFT [32]. However, each of these indices carries inherent constraints. Kaolin intake in mice is characteristically unstable across strains and individuals [21], and this limitation is particularly pronounced in the C57BL/6 strain. Moreover, the gold‐standard status of pica as an MS indicator was originally established in rats [12], and significant species differences exist between rats and mice in pica expression. Additionally, kaolin intake primarily reflects gastrointestinal distress rather than the disabling spatial and motor deficits central to the clinical syndrome. The MS index has an upper limit; once the intestinal contents are completely emptied, the number of bowel movements no longer increases with the degree of peristalsis [33]. Similarly, OFT locomotor counts are susceptible to anxiety‐related suppression and lack the spatial resolution to assess navigation impairment [34, 35]. Given these limitations, we turned to the CatWalk gait analysis system as a complementary, high‐resolution tool for quantifying MS‐related motor deficits. MS mice exhibited pronounced disruptions in motor coordination, postural stability, and gait rhythm. Reduced paw contact duration, accelerated paw detachment, and diminished contact intensity and support force reflected impaired fine regulation of limb loading and sensorimotor feedback, indicative of limb ataxia [36, 37]. Disordered step sequences signified disrupted temporal control of gait and diminished interlimb coordination [37]. The narrowed distance between ipsilateral fore‐ and hind‐paws represented a compensatory adjustment to minimize center of mass displacement [38]. These phenotypes represent typical motor behavioral features of MS resulting from disrupted vestibular spatial signal transmission and integration. Operationally simple, highly sensitive, and temporally resolved, the CatWalk platform can be readily integrated with complementary assays to provide a robust behavior‐based framework for MS detection.

Sexual dimorphism was also evident. Female mice showed no significant behavioral abnormalities under identical stimulation, suggesting relative resilience to MS. This finding appears inconsistent with clinical epidemiological reports of higher MS susceptibility in human females [39]. The mechanisms underlying this apparent species divergence remain unclear, and we did not directly assess hormonal influences in the present study. Nevertheless, this observation raises the possibility that sex‐dependent regulation of the CLA–POR circuit may contribute to differential MS susceptibility. Notably, the MS index—an index of visceral/autonomic responses to vestibular conflict—showed a trend toward an increase in female mice (*p* = 0.0570), whereas spatial and motor performance remained intact. This dissociation suggests that the observed sexual dimorphism resides downstream of the vestibular–brainstem autonomic axis, potentially at the level of the CLA–POR circuit that gates spatial orientation and motor coordination. If confirmed, this would further support a specific role of the CLA–POR pathway in the spatial‐motor rather than the autonomic dimension of MS. Given that sex differences are increasingly recognized as an important biological variable in neuroscience, future studies systematically examining estrous cycle stage, hormonal status, and sex‐specific CLA–POR circuit activity will be necessary to elucidate the mechanisms underlying this sexual dimorphism.

Previous MS research has focused predominantly on canonical subcortical pathways, including the brainstem, vestibular system, cerebellar flocculus, and reticular formation [40, 41, 42]. Although recent work has begun to implicate cortical regions, attention has largely centered on areas such as the mPFC, while the CLA has remained underexplored [5, 6]. The present study identifies the CLA–POR pathway as a higher‐order cortical modulator of MS. This organization positions the CLA as a multimodal integration hub that transmits conflict signals to the POR and downstream hippocampal–entorhinal system [9, 43]. The present study establishes a functional link between the CLA–POR pathway and MS pathophysiology.

The present findings should be considered within the broader context of the vestibular cortical network and established MS‐related brain regions. The vestibular system transmits ascending signals through two major pathways: a subcortical route involving the vestibular nuclei and thalamus, and a cortical route engaging the insular cortex (IC) and parieto‐insular vestibular cortex (PIVC) [44, 45]. Our observation of increased FOS expression in the MVePC and thalamic regions (Figure S4A,B) is consistent with the established role of these structures in vestibular processing. The CLA is among the most extensively connected forebrain structures [7, 8], although direct monosynaptic input from the vestibular nuclei to the CLA remains incompletely established; its anatomical position within insula–thalamus–hippocampal spatial networks places it in a strong position to integrate vestibular conflict signals with visuospatial and proprioceptive information [46]. The POR, in turn, constitutes a component of the medial temporal lobe spatial navigation system, with direct connections to the hippocampal‐entorhinal network [47]. This anatomical architecture places the CLA–POR pathway at the interface between vestibular processing and spatial navigation, providing a plausible circuit substrate for the spatial disorientation that characterizes MS.

The relationship between the CLA–POR pathway and other established MS‐related regions warrants specific consideration. The vestibular nuclei provide ascending drive to the thalamus [48], which in turn innervates the CLA and POR. This pathway may represent the route through which vestibular conflict signals reach the CLA–POR circuit. The parabrachial nucleus (PBN), a key relay for visceral malaise signals, receives vestibular input and may contribute to the autonomic symptoms of MS [49]. Our finding that CLA manipulation did not affect pica behavior suggests that the CLA–POR pathway is more specifically engaged in spatial and motor deficits rather than visceral discomfort. CLA does not project directly to the cerebellum, but it may influence cerebellar function indirectly through its projections to the POR and other cortical areas that innervate the pontine nuclei. The IC, a core vestibular cortical region [44], has connections with the CLA [50], suggesting that CLA may serve as a hub integrating insular vestibular signals with hippocampal spatial information. Collectively, these considerations position the CLA–POR pathway as a higher‐order integration node within the multisensory network underlying MS, complementing rather than replacing the established brainstem‐centric model.

At the cellular level, MS induced a marked shift in the E/I balance within the CLA–POR circuit. Chemogenetic rescue experiments confirmed the causal role of this E/I imbalance. Activation of CLA^VGluT2^–POR projections restored spatial orientation in the Morris water maze without altering gait parameters, whereas inhibition of this pathway neither exacerbated MS nor produced adverse effects in healthy mice. Conversely, inhibition of CLA^GAD67^–POR projections ameliorated both spatial memory deficits and motor coordination abnormalities on the CatWalk, while activation of this pathway was similarly inert under both pathological and baseline conditions. Consistent with the behavioral rescue, we next examined whether manipulation of CLA projection neurons alters downstream POR neuronal activity. Activation of CLA^VGluT2^–POR projections increased FOS expression in POR, whereas inhibition of CLA^GAD67^–POR projections similarly restored POR FOS expression, demonstrating that manipulation of CLA projection neurons alters downstream POR neuronal activity in a manner consistent with the behavioral rescue. These data provide a functional link between CLA E/I shift and POR activity; the direction of this alteration matches the behavioral rescue, thereby completing a CLA (manipulation)–POR (activity change)–behavior (rescue) chain. Moreover, fiber photometry recordings performed immediately after the cessation of rotation further corroborated the E/I imbalance within the CLA–POR pathway, demonstrating that this shift represents a sustained alteration in circuit function rather than a transient motion artifact. This temporally resolved readout offers functional evidence that is complementary to the endpoint FOS and chemogenetic findings. These bidirectional manipulations rescued pathological deficits without disrupting normal function, underscoring the therapeutic potential of the CLA‐centered circuit. Notably, the two pathways exhibited functional specialization: glutamatergic inputs preferentially supported spatial cognition, whereas GABAergic inputs gated motor coordination, a segregation mirrored by their divergent axonal morphologies. The differential behavioral outcomes of these two manipulations—with GAD67^+^ inhibition improving both spatial memory and gait, whereas VGluT2^+^ activation improved only spatial memory—may reflect recruitment of distinct downstream circuits within the POR or divergent engagement of downstream targets beyond the POR. Alternatively, these differences could arise from distinct temporal progression or magnitudes of modulation produced by each manipulation. Our findings differ from previous studies on MS‐related neuronal subtypes, which have primarily focused on excitatory neurons [51, 52], and open new avenues for exploring bidirectional E/I regulation in MS.

These findings align with the sensory conflict theory: when actual sensory signals conflict with vestibular‐derived predictions, the CLA–POR circuit normally encodes and resolves such prediction errors [3]. Under MS, however, the vestibular system sends excitatory signals to the thalamus [23], which indirectly disrupts CLA–POR E/I balance and prevents effective conflict resolution. The observation that further inhibition of an already suppressed CLA^VGluT2^–POR pathway or activation of an already enhanced CLA^GAD67^–POR pathway produced no additional behavioral effects supports the interpretation that these neurons operate near pathological extremes in MS, leaving little dynamic range for exacerbation. This circuit‐relevant vulnerability suggests that pharmacological or neuromodulatory strategies aimed at restoring CLA–POR E/I balance, either by enhancing glutamatergic excitation or suppressing GABAergic inhibition, may offer a safe and effective approach for MS intervention. However, several limitations should be considered when interpreting these findings. Given the broad projections of CLA neurons to multiple cortical and subcortical targets, the observed behavioral effects may reflect modulation of multiple CLA output pathways rather than the CLA–POR pathway alone. While our chemogenetic manipulations demonstrate that the CLA–POR circuit is a critical functional node in the manifestation of MS‐related behaviors, we acknowledge that the current data cannot definitively determine whether the observed E/I shift represents the primary trigger or a downstream consequence of altered vestibular‐thalamic input. The robust bidirectional rescue of behavioral deficits by CLA manipulation, however, suggests that this circuit is not merely a passive readout but rather an active and necessary relay in the pathological cascade. Future studies employing temporally precise interventions, such as optogenetic silencing during rotation, will be essential to establish the hierarchical sequence of events. Furthermore, whether the CLA–POR circuit is specifically engaged in MS pathophysiology or instead serves a broader role in spatial cognition and motor control—with its involvement in MS representing a secondary consequence—remains to be determined. Due to the technical constraints imposed by the high‐speed rotation apparatus, fiber photometry recordings were performed immediately (within 10 min) after the cessation of rotation rather than during the rotation protocol itself. This approach captured the persistent post‐stimulation state of CLA neuronal activity but did not resolve the online dynamics during the actual sensory conflict. Future studies employing miniature recording systems compatible with the rotation apparatus will be necessary to capture the real‐time dynamics of CLA activity during MS induction. Behavioral assessments were conducted immediately after the final of four consecutive daily rotations, within the acute post‐rotation window during which CLA/POR FOS suppression is observed. At the time of testing, therefore, the circuit was in the suppressed state. Notably, the progressive exacerbation of behavioral deficits across repeated rotations, in contrast to the rapid recovery of FOS expression, suggests that sustained behavioral impairment may involve cumulative or plasticity‐related mechanisms that outlast the immediate FOS response—a hypothesis that warrants further investigation. At the molecular level, our study did not examine the molecular basis underlying the MS‐induced E/I shift. Future studies investigating changes in glutamatergic and GABAergic receptor expression, synaptic protein composition, and neurotransmitter release dynamics will be necessary to elucidate the plasticity mechanisms that mediate this functional remodeling of the CLA–POR circuit. In addition, given that CaMKII^+^ neurons constitute the largest neuronal population in the CLA [53], future studies should also consider modulating the CLA^CaMKII^–POR pathway to examine its effects on regulating MS. The present study focused on the upstream CLA component of this pathway and did not directly assess POR neuronal activity or its downstream functional contributions; investigating POR neuronal dynamics and their behavioral relevance therefore constitutes a critical avenue for future research. Future studies employing pathway‐specific manipulations, such as terminal‐level inhibition or intersectional genetic strategies, will be necessary to dissociate the contributions of individual CLA output pathways and to establish the specificity of this circuit to MS. Additionally, the translational potential of chemogenetic approaches faces significant challenges. These include the safety concerns associated with viral vector delivery in humans, potential off‐target effects of CNO and its metabolites [54], the long‐term safety of DREADD expression, and the difficulty of achieving cell‐type‐specific targeting in humans. Alternative therapeutic strategies that restore CLA–POR E/I balance—such as pharmacological modulation of glutamatergic or GABAergic signaling, or non‐invasive brain stimulation techniques—may offer more feasible clinical pathways, although these remain speculative at present, and validation in non‐human primates constitutes an essential prerequisite for any clinical translation. Finally, all experiments were conducted in a single species (C57BL/6 mice). While the mouse model offers significant advantages for circuit‐level manipulation, the anatomical structure of the mouse CLA differs from that of non‐human primates [18], particularly in terms of relative size and cortical connectivity. Moreover, species differences in MS susceptibility and behavioral expression may limit the generalizability of our findings. Future studies using non‐human primates will be essential to validate the translational relevance of the CLA–POR circuit.

## 5 Conclusion

The present study demonstrates that CLA projection neurons represent a potential target for MS intervention. Unlike brainstem‐targeted antiemetics that cause sedation or cognitive side effects, modulation of cortical E/I balance may alleviate spatial disorientation while preserving alertness. The present chemogenetic rescue experiments provide proof‐of‐concept that bidirectional tuning of the CLA–POR pathway is sufficient to ameliorate MS symptoms without affecting baseline behavior, supporting a favorable therapeutic window.

## Author Contributions

**Wen‐Rui Jiang:** writing – original draft, visualization, investigation, formal analysis, data curation, conceptualization; **Xin‐Tong Qiu:** investigation, formal analysis, conceptualization; **Zi‐Yi Yang:** investigation – surgery, investigation – immunohistochemical staining, investigation – FISH; **Yu‐Tong Guo:** investigation – FISH, investigation – behavioral observation; **Zi‐Yan Wang:** investigation – behavioral observation; **Jia‐Rui Xu:** investigation – surgery; **Lu Yang:** investigation – behavioral observation; **Zhong‐Xuan Du:** investigation – behavioral observation; **Xiao‐Tian Yang:** investigation – immunohistochemical staining; **Tian‐Yu Zhao:** formal analysis, data curation; **Fu‐Xing Zhang:** conceptualization; **Rui‐Chao Gou:** investigation – MS provocation; **Pan Wang:** writing – original draft, supervision, project administration, funding acquisition, conceptualization; **Ming‐Ming Zhang:** writing – review and editing, visualization, supervision, project administration, funding acquisition, formal analysis, conceptualization. All authors have read and agreed to the published version of the manuscript.

## Funding

This work was supported by grants from the Brain Science and Brain‐like Intelligence Technology—National Science and Technology Major Project (No. 2021ZD0204403 to M.‐M. Zhang and F.‐X. Zhang), the National Natural Science Foundation of China (No. 82371246 to M.‐M. Zhang), the Outstanding Youth Funding of Shaanxi Province (No. 2024JC‐JCQN‐74 to M.‐M. Zhang), and the Natural Science Basic Research Program of Shaanxi Province (No. 2025JC‐YBMS‐940 to P. Wang).

## Ethics Statement

All procedures complied with institutional and national guidelines and were approved by the Animal Care and Use Committee of the Fourth Military Medical University (IACUC‐20211018).

## Conflicts of Interest

The authors declare no conflicts of interest.

## Supporting information


**Figure S1:** The detailed experimental timeline for motion sickness (MS) model. (A) Experimental timeline.
**Figure S2:** MS model lacks efficacy in female mice. (A) Average kaolin intake in mice (*n* = 6 mice per group). (B) Representative activity track maps of spontaneous activity from the indicated groups. (C) Total distance, central distance (%), and central time for open field test (OFT) in mice (*n* = 6 mice per group). (D) Representative activity track maps of Morris water maze from the indicated groups. (E) Platform crossing times, effective distance, effective time, and effective distance (%) for Morris water maze in mice (*n* = 6 mice per group). (F) The print view and footfall patterns of the step sequence in a mouse that passes through the glass pane. AB, alternate pattern (left front [LF]–right hind [RH]–right front [RF]–left hind [LH]). AA, alternate pattern (RF–RH–LF–LH). CA, cruciate pattern (RF–LF–RH–LH). CB, cruciate pattern (LF–RF–LH–RH). (G) RH stand, RH stand index, RH mean intensity, Regularity index, Print Positions right paws, and Base of Support hind paws for CatWalk gait analysis in mice (*n* = 6 mice per group). Data are mean ± SEM; statistical analyses were conducted using unpaired *t*‐test. Statistical details are provided in Table S1. Ctrl, control; LF, left front; LH, left hind; MS, motion sickness; RF, right front; RH, right hind.
**Figure S3:** Total distance of Morris water maze and MS index between MS group and the control group. (A) Total distance of Morris water maze in male mice (*n* = 6 mice per group). (B) MS index in male mice (*n* = 6 mice per group). (C) Total distance of Morris water maze in female mice (*n* = 6 mice per group). (D) MS index in female mice (*n* = 6 mice per group). Data are mean ± SEM; statistical analyses were conducted using unpaired *t*‐test. Statistical details are provided in Table S1. Ctrl, control; MS, motion sickness.
**Figure S4:** FOS expression in MS mice. (A) Bidirectional horizontal bar chart with group backgrounds showing the number of FOS^+^ neurons in different brain regions. The number in each region is indicated on the side of the bar chart. (B) Quantification of FOS^+^ cell density expressed as MS group percentage change from Ctrl group. Representative immunofluorescent photos of FOS immunoreactive (green) in the medial vestibular nucleus, parvicellular part (MVePC) (C), claustrum (CLA) (E), and postrhinal cortex (POR) (G). The average number of FOS labeling neurons in the MVePC (*n* = 6 sections/3 mice per group) (D), CLA (*n* = 6 sections/3 mice per group) (F), and POR (*n* = 6 sections/3 mice per group) (H) from the indicated groups. Scale bar, 100 μm. Data are mean ± SEM; statistical analyses were conducted using two‐way mixed‐effects ANOVA; *p < 0.05, **p < 0.01, ***p < 0.001. Statistical details are provided in Table S1. CLA, claustrum; Ctrl, control; MS, motion sickness; MVePC, medial vestibular nucleus, parvicellular part; POR, postrhinal cortex.
**Figure S5:** Validation of the mutual exclusivity of vesicular glutamate transporter 2^+^ (VGluT2) and glutamic acid decarboxylase 67^+^ (GAD67) CLA → POR projection neurons. (A) Confocal fluorescence in situ hybridization (FISH) combined with immunofluorescence staining images showing VGluT2 mRNA^+^ (green), beads^+^ (red), and GAD67^+^ neurons (gray) in the CLA with DAPI (blue)‐counterstained. Scale bar, 400 μm. (B) Venn diagram of the proportional distribution of retrogradely labeled CLA‐POR neurons, the percentage is expressed as the ratio of double labeled neurons to retrograde labeled neurons (*n* = 6 sections/3 mice). Dashed lines delineate brain region boundaries; solid boxes indicate magnified areas. Arrows indicate VGluT2 mRNA^+^/Beads^+^ (white), GAD67^+^/Beads^+^ (yellow) or triple‐labeled (blue) neurons. GAD67, glutamic acid decarboxylase 67; VGluT2, vesicular glutamate transporter 2.
**Figure S6:** Long‐range projections from the CLA to POR. (A) Schematic of viral injection of AAV2/9‐hEF1a‐DIO‐hChR2(H134R)‐mCherry into the CLA, and whole‐cell patch recording in POR neurons. (B) Representative confocal image showing injection site for mCherry^+^ expression in the CLA. (C) Representative confocal image showing mCherry^+^ axonal fibers in the POR. (D) Fluorescent image showing recorded neurons labeled with mCherry and biocytin (green) in the POR. (E) Representative traces showing that optogenetically evoked postsynaptic currents (EPSCs) in POR neurons. (F) Schematic of viral injection of AAV2/9‐hEF1a‐DIO‐hChR2(H134R)‐mCherry into the CLA, and whole‐cell patch recording in POR neurons. (G) Representative confocal image showing injection site for mCherry^+^ expression in the CLA. (H) Representative confocal image showing mCherry^+^ axonal fibers in the POR. (I) Fluorescent image showing recorded neurons labeled with mCherry and biocytin (green) in the POR. (J) Representative traces showing that optogenetically evoked control postsynaptic currents (IPSCs) (purple), after IPSCs after adding Gabazine (black), and IPSCs after adding CNQX + AP5 (green) in POR neurons. Scale bar, 200 μm (B, C, G, H), 10 μm (D, I). CLA, claustrum; POR, postrhinal cortex; VGAT, vesicular GABA transporter; VGluT2, vesicular glutamate transporter 2.
**Figure S7:** Chemogenetic inhibition of the CLA^VGluT2^–POR pathway in MS mice. (A) Schematic of viral injection of AAV2/9‐EF1a‐DIO‐hM4D(Gi)‐mCherry or AAV2/9‐EF1a‐DIO‐mCherry into the CLA, and rAAV2/Retro‐VGluT2‐SV40 NLS‐Cre into the POR. (B) Representative confocal image showing injection site for hM4D(Gi) expression in the CLA with DAPI‐counterstained. Scale bar, 100 μm. (C) Representative activity track maps of spontaneous activity from the indicated groups. (D) Total distance, central distance (%), and central time for OFT in mice (*n* = 6 mice per group). (E) Representative activity track maps of Morris water maze from the indicated groups. (F) Platform crossing times, effective distance, effective time, and effective distance (%) for Morris water maze in mice (*n* = 8 mice in mCherry group, *n* = 6 mice in hM4Di group). (G) The print view and footfall patterns of the step sequence in a mouse that passes through the glass pane. CB, cruciate pattern (LF–RF–LH–RH). CA, cruciate pattern (RF–LF–RH–LH). AA, alternate pattern (RF–RH–LF–LH). (H) RH stand, RH stand index, RH mean intensity, Regularity index, Print Positions right paws, and Base of Support hind paws for CatWalk gait analysis in mice (*n* = 8 mice per group). Data are mean ± SEM; statistical analyses were conducted using unpaired *t*‐test. Statistical details are provided in Table S1. CLA, claustrum; LF, left front; LH, left hind; POR, postrhinal cortex; RF, right front; RH, right hind.
**Figure S8:** Chemogenetic activation of the CLA^GAD67^–POR pathway in MS mice. (A) Schematic of viral injection of AAV2/9‐GAD67‐DIO‐hM3D(Gq)‐mCherry or AAV2/9‐GAD67‐DIO‐mCherry into the CLA, and rAAV2/Retro‐hSyn‐CRE into the POR. (B) Representative confocal image showing injection site for hM3D(Gq) expression in the CLA with DAPI‐counterstained. Scale bar, 100 μm. (C) Representative activity track maps of spontaneous activity from the indicated groups. (D) Total distance, central distance (%), and central time for OFT in mice (*n* = 6 mice per group). (E) Representative activity track maps of Morris water maze from the indicated groups. (F) Platform crossing times, effective distance, effective time, and effective distance (%) for Morris water maze in mice (*n* = 6 mice per group). (G) The print view and footfall patterns of the step sequence in a mouse that passes through the glass pane. CA, cruciate pattern (RF–LF–RH–LH). AA, alternate pattern (RF–RH–LF–LH). CB, cruciate pattern (LF–RF–LH–RH). (H) RH stand, RH stand index, RH mean intensity, Regularity index, Print Positions right paws, and Base of Support hind paws for CatWalk gait analysis in mice (*n* = 6 mice per group). Data are mean ± SEM; statistical analyses were conducted using unpaired *t*‐test. Statistical details are provided in Table S1. CLA, claustrum; LF, left front; LH, left hind; POR, postrhinal cortex; RF, right front; RH, right hind.
**Figure S9:** Chemogenetic inhibition of the CLA^VGluT2^–POR pathway in C57 mice. (A) Schematic of viral injection of AAV2/9‐EF1a‐DIO‐hM4D(Gi)‐mCherry or AAV2/9‐EF1a‐DIO‐mCherry into the CLA, and rAAV2/Retro‐VGluT2‐SV40 NLS‐Cre into the POR. (B) Representative confocal image showing injection site for hM4D(Gi) expression in the CLA with DAPI‐counterstained. Scale bar, 100 μm. (C) Representative activity track maps of spontaneous activity from the indicated groups. (D) Total distance, central distance (%), and central time for OFT in mice (*n* = 6 mice per group). (E) Representative activity track maps of Morris water maze from the indicated groups. (F) Platform crossing times, effective distance, effective time, and effective distance (%) for Morris water maze in mice (*n* = 6 mice per group). (G) The print view and footfall patterns of the step sequence in a mouse that passes through the glass pane. CB, cruciate pattern (LF–RF–LH–RH). AA, alternate pattern (RF–RH–LF–LH). (H) RH stand, RH stand index, RH mean intensity, Regularity index, Print Positions right paws, and Base of Support hind paws for CatWalk gait analysis in mice (*n* = 6 mice per group). Data are mean ± SEM; statistical analyses were conducted using unpaired *t*‐test. Statistical details are provided in Table S1. CLA, claustrum; LF, left front; LH, left hind; POR, postrhinal cortex; RF, right front; RH, right hind.
**Figure S10:** Chemogenetic activation of the CLA^GAD67^–POR pathway in C57 mice. (A) Schematic of viral injection of AAV2/9‐GAD67‐DIO‐hM3D(Gq)‐mCherry or AAV2/9‐GAD67‐DIO‐mCherry into the CLA, and rAAV2/Retro‐hSyn‐CRE into the POR. (B) Representative confocal image showing injection site for hM3D(Gq) expression in the CLA with DAPI‐counterstained. Scale bar, 100 μm. (C) Representative activity track maps of spontaneous activity from the indicated groups. (D) Total distance, central distance (%), and central time for OFT in mice (*n* = 6 mice per group). (E) Representative activity track maps of Morris water maze from the indicated groups. (F) Platform crossing times, effective distance, effective time, and effective distance (%) for Morris water maze in mice (*n* = 6 mice per group). (G) The print view and footfall patterns of the step sequence in a mouse that passes through the glass pane. CB, cruciate pattern (LF–RF–LH–RH). AA, alternate pattern (RF–RH–LF–LH). AB, alternate pattern (LF–RH–RF–LH). CA, cruciate pattern (RF–LF–RH–LH). (H) RH stand, RH stand index, RH mean intensity, Regularity index, Print Positions right paws, and Base of Support hind paws for CatWalk gait analysis in mice (*n* = 6 mice per group). Data are mean ± SEM; statistical analyses were conducted using unpaired *t*‐test. Statistical details are provided in Table S1. CLA, claustrum; LF, left front; LH, left hind; POR, postrhinal cortex; RF, right front; RH, right hind.
**Figure S11:** Chemogenetic activation of the CLA^VGluT2^–POR pathway alleviates MS‐like behaviors, experimental group received CNO + hM3Dq, control group received Saline + hM3Dq. (A) Schematic of viral injection of AAV2/9‐EF1a‐DIO‐hM3D(Gq)‐mCherry into the CLA, and rAAV2/Retro‐VGluT2‐SV40 NLS‐Cre into the POR. (B) Representative confocal image showing injection site for hM3D(Gq) expression in the CLA with DAPI‐counterstained. Scale bar, 100 μm. (C) Representative activity track maps of spontaneous activity from the indicated groups. (D) Total distance, central distance (%), and central time for OFT in mice (*n* = 6 mice per group). (E) Representative activity track maps of Morris water maze from the indicated groups. (F) Platform crossing times, effective distance, effective time, and effective distance (%) for Morris water maze in mice (*n* = 6 mice in Saline group, *n* = 10 mice in clozapine N‐oxide [CNO] group). (G) The print view and footfall patterns of the step sequence in a mouse that passes through the glass pane. AA, alternate pattern (RF–RH–LF–LH). AB, alternate pattern (LF–RH–RF–LH). CA, cruciate pattern (RF–LF–RH–LH). (H) RH stand, RH stand index, RH mean intensity, Regularity index, Print Positions right paws, and Base of Support hind paws for CatWalk gait analysis in mice (*n* = 6 mice in Saline group, *n* = 8 mice in CNO group). Data are mean ± SEM; statistical analyses were conducted using unpaired t‐test; *p < 0.05, **p < 0.01, ***p < 0.001. Statistical details are provided in Table S1. CLA, claustrum; CNO, clozapine N‐oxide; LF, left front; LH, left hind; POR, postrhinal cortex; RF, right front; RH, right hind.
**Figure S12:** Chemogenetic inhibition of the CLA^GAD67^–POR pathway alleviates MS‐like behaviors, experimental group received CNO + hM4Di, control group received Saline + hM4Di. (A) Schematic of viral injection of AAV2/9‐GAD67‐DIO‐hM4D(Gi)‐mCherry into the CLA, and rAAV2/Retro‐hSyn‐CRE into the POR. (B) Representative confocal image showing injection site for hM4D(Gi) expression in the CLA with DAPI‐counterstained. Scale bar, 100 μm. (C) Representative activity track maps of spontaneous activity from the indicated groups. (D) Total distance, central distance (%), and central time for OFT in mice (*n* = 6 mice per group). (E) Representative activity track maps of Morris water maze from the indicated groups. (F) Platform crossing times, effective distance, effective time, and effective distance (%) for Morris water maze in mice (*n* = 6 mice in Saline group, *n* = 7 mice in CNO group). (G) The print view and footfall patterns of the step sequence in a mouse that passes through the glass pane. AA, alternate pattern (RF–RH–LF–LH). CB, cruciate pattern (LF–RF–LH–RH). AB, alternate pattern (LF–RH–RF–LH). (H) RH stand, RH stand index, RH mean intensity, Regularity index, Print Positions right paws, and Base of Support hind paws for CatWalk gait analysis in mice (*n* = 6 mice in Saline group, *n* = 8 mice in CNO group). Data are mean ± SEM; statistical analyses were conducted using unpaired *t*‐test; *p < 0.05, **p < 0.01, ***p < 0.001, ****p < 0.0001. Statistical details are provided in Table S1. CLA, claustrum; CNO, clozapine N‐oxide; LF, left front; LH, left hind; POR, postrhinal cortex; RF, right front; RH, right hind.
**Figure S13:** Representative injection sites at multiple anterior–posterior levels. (A–G) Different representative injection sites at multiple anterior–posterior levels in the CLA. Dashed lines delineate brain region boundaries. Scale bar, 400 μm. CLA, claustrum; LXN, latexin; POR, postrhinal cortex.
**Figure S14:** FOS expression in the POR following chemogenetic manipulation of CLA‐POR projections in MS mice. (A) Confocal images showing the FOS immunoreactive (green) in the POR in mCherry group in the CLA^VGluT2^–POR pathway. (B) Confocal images showing the FOS immunoreactive (green) in the POR in hM3Dq group in the CLA^VGluT2^–POR pathway. (C) The FOS^+^ neurons in the POR (*n* = 6 sections/3 mice per group). (D) Confocal images showing the FOS immunoreactive (green) in the POR in mCherry group in the CLA^GAD67^–POR pathway. (E) Confocal images showing the FOS immunoreactive (green) in the POR in hM4Di group in the CLAGAD67–POR pathway. (F) The FOS^+^ neurons in the POR (*n* = 6 sections/3 mice per group). White dashed line, the extent of the brain region. White solid lines, the magnified regions. Scale bar, 100 μm. Data are mean ± SEM; statistical analyses were conducted using unpaired *t*‐test; **p < 0.01, ***p < 0.001. Statistical details are provided in Table S1. CLA, claustrum; POR, postrhinal cortex.
**Figure S15:** MS stimuli change excitability in the CLA–POR pathway. (A) Schematic of viral injection of AAV2/9‐EF1a‐DIO‐GCaMP6s into the CLA, and AAV2/Retro‐mVGLUT2‐Cre into the POR, and optical fiber implantation in the CLA. (B) Fluorescence image showing viral expression and the trace of optical fiber implantation within the CLA. (C–E) Heatmap, averaged ΔF/F traces and area under the curve for calcium responses to control group and MS group (*n* = 8 per group, one data point per mouse). (F) Schematic of viral injection of AAV2/9‐EF1a‐DIO‐GCaMP6s into the CLA, and AAV2/Retro‐mVGAT1‐Cre into the POR, and optical fiber implantation in the CLA. (G) Fluorescence image showing viral expression and the trace of optical fiber implantation within the CLA. (H–J) Heatmap, averaged ΔF/F traces and area under the curve for calcium responses to control group and MS group (*n* = 6 per group, one data point per mouse). Scale bar, 200 μm. Dashed lines delineate brain region boundaries; solid lines indicate fiber implantation boundaries. Data are mean ± SEM; statistical analyses were conducted using unpaired *t*‐test; *p < 0.05. Statistical details are provided in Table S1. CLA, claustrum; Ctrl, control; MS, motion sickness; POR, postrhinal cortex.
**Table S1:** Detailed statistical results for all comparisons.

## Data Availability

The data that support the findings of this study are available from the corresponding author upon reasonable request.
