# Supplementary material for: Bidirectional Modulation of the Claustrum–Postrhinal Cortex Circuit Alleviates Motion Sickness in Mice: A Cortical Target for Sensory Conflict Resolution
Source: CNS Neurosci Ther. 2026 Nov 12;32(11):e71261. doi: 10.1002/cns.71261 (PMC13757399; doi:10.1002/cns.71261)
Supplement: Supplementary file 1 [file CNS-32-e71261-s001.docx]

**FIGURE S1** The detailed experimental timeline for motion sickness (MS) model. (A) Experimental timeline.

**FIGURE S2** MS model lacks efficacy in female mice. (A) Average kaolin intake in mice (n = 6 mice per group). (B) Representative activity track maps of spontaneous activity from the indicated groups. (C) Total distance, central distance (%), and central time for open field test (OFT) in mice (n = 6 mice per group). (D) Representative activity track maps of Morris water maze from the indicated groups. (E) Platform crossing times, effective distance, effective time, and effective distance (%) for Morris water maze in mice (n = 6 mice per group). (F) The print view and footfall patterns of the step sequence in a mouse that passes through the glass pane. AB, alternate pattern [left front (LF)-right hind (RH)-right front (RF)-left hind (LH)]. AA, alternate pattern (RF-RH-LF-LH). CA, cruciate pattern (RF-LF-RH-LH). CB, cruciate pattern (LF-RF-LH-RH). (G) RH stand, RH stand index, RH mean intensity, Regularity index, Print Positions right paws, and Base of Support hind paws for CatWalk gait analysis in mice (n = 6 mice per group). Data are mean ± SEM; statistical analyses were conducted using unpaired *t* test. Statistical details are provided in Supplementary Table S1. Ctrl, control. LF, left front. LH, left hind. MS, motion sickness. RF, right front. RH, right hind.

**FIGURE S3** Total distance of Morris water maze and MS index between MS group and the control group. (A) Total distance of Morris water maze in male mice (n = 6 mice per group). (B) MS index in male mice (n = 6 mice per group). (C) Total distance of Morris water maze in female mice (n = 6 mice per group). (D) MS index in female mice (n = 6 mice per group). Data are mean ± SEM; statistical analyses were conducted using unpaired *t* test. Statistical details are provided in Supplementary Table S1. Ctrl, control. MS, motion sickness.

**FIGURE S4** FOS expression in MS mice. (A) Bidirectional horizontal bar chart with group backgrounds showing the number of FOS^+^ neurons in different brain regions. The number in each region is indicated on the side of the bar chart. (B) Quantification of FOS⁺ cell density expressed as MS group percentage change from Ctrl group. Representative immunofluorescent photos of FOS immunoreactive (green) in the medial vestibular nucleus, parvicellular part (MVePC) (C), claustrum (CLA) (E), and postrhinal cortex (POR) (G). The average number of FOS labeling neurons in the MVePC (n = 6 sections/3 mice per group) (D), CLA (n = 6 sections/3 mice per group) (F), and POR (n = 6 sections/3 mice per group) (H) from the indicated groups. Scale bar, 100 µm. Data are mean ± SEM; statistical analyses were conducted using two-way mixed-effects ANOVA; **p <* 0.05, ***p <* 0.01, ****p <* 0.001. Statistical details are provided in Supplementary Table S1. CLA, claustrum. Ctrl, control. MS, motion sickness. MVePC, medial vestibular nucleus, parvicellular part. POR, postrhinal cortex.

**FIGURE S5** Validation of the mutual exclusivity of vesicular glutamate transporter 2⁺ (VGluT2) and glutamic acid decarboxylase 67⁺ (GAD67) CLA→POR projection neurons. (A) Confocal fluorescence *in situ* hybridization (FISH) combined with immunofluorescence staining images showing VGluT2 mRNA⁺ (green), beads⁺ (red), and GAD67^+^ neurons (gray) in the CLA with DAPI (blue)-counterstained. Scale bar, 400 µm. (B) Venn diagram of the proportional distribution of retrogradely labeled CLA-POR neurons, the percentage is expressed as the ratio of double labeled neurons to retrograde labeled neurons (n = 6 sections/3 mice). Dashed lines delineate brain region boundaries; solid boxes indicate magnified areas. Arrows indicate VGluT2 mRNA⁺/Beads⁺ (white), GAD67⁺/Beads⁺ (yellow) or triple-labeled (blue) neurons. GAD67, glutamic acid decarboxylase 67. VGluT2, vesicular glutamate transporter 2.

**FIGURE S6** Long-range projections from the CLA to POR. (A) Schematic of viral injection of AAV2/9-hEF1a-DIO-hChR2(H134R)-mCherry into the CLA, and whole-cell patch recording in POR neurons. (B) Representative confocal image showing injection site for mCherry^+^ expression in the CLA. (C) Representative confocal image showing mCherry^+^ axonal fibers in the POR. (D) Fluorescent image showing recorded neurons labeled with mCherry and biocytin (green) in the POR. (E) Representative traces showing that optogenetically evoked postsynaptic currents (EPSCs) in POR neurons. (F) Schematic of viral injection of AAV2/9-hEF1a-DIO-hChR2(H134R)-mCherry into the CLA, and whole-cell patch recording in POR neurons. (G) Representative confocal image showing injection site for mCherry^+^ expression in the CLA. (H) Representative confocal image showing mCherry^+^ axonal fibers in the POR. (I) Fluorescent image showing recorded neurons labeled with mCherry and biocytin (green) in the POR. (J) Representative traces showing that optogenetically evoked control postsynaptic currents (IPSCs) (purple), after IPSCs after adding Gabazine (black), and IPSCs after adding CNQX + AP5 (green) in POR neurons. Scale bar, 200 µm (B, C, G, H), 10 µm (D, I). CLA, claustrum. POR, postrhinal cortex. VGAT, vesicular GABA transporter. VGluT2, vesicular glutamate transporter 2.

**FIGURE S7** Chemogenetic inhibition of the CLA^VGluT2^-POR pathway in MS mice. (A) Schematic of viral injection of AAV2/9-EF1a-DIO-hM4D(Gi)-mCherry or AAV2/9-EF1a-DIO-mCherry into the CLA, and rAAV2/Retro-VGluT2-SV40 NLS-Cre into the POR. (B) Representative confocal image showing injection site for hM4D(Gi) expression in the CLA with DAPI-counterstained. Scale bar, 100 µm. (C) Representative activity track maps of spontaneous activity from the indicated groups. (D) Total distance, central distance (%), and central time for OFT in mice (n = 6 mice per group). (E) Representative activity track maps of Morris water maze from the indicated groups. (F) Platform crossing times, effective distance, effective time, and effective distance (%) for Morris water maze in mice (n = 8 mice in mCherry group, n = 6 mice in hM4Di group). (G) The print view and footfall patterns of the step sequence in a mouse that passes through the glass pane. CB, cruciate pattern (LF-RF-LH-RH). CA, cruciate pattern (RF-LF-RH-LH). AA, alternate pattern (RF-RH-LF-LH). (H) RH stand, RH stand index, RH mean intensity, Regularity index, Print Positions right paws, and Base of Support hind paws for CatWalk gait analysis in mice (n = 8 mice per group). Data are mean ± SEM; statistical analyses were conducted using unpaired *t* test. Statistical details are provided in Supplementary Table S1. CLA, claustrum. LF, left front. LH, left hind. POR, postrhinal cortex. RF, right front. RH, right hind.

**FIGURE S8** Chemogenetic activation of the CLA^GAD67^-POR pathway in MS mice. (A) Schematic of viral injection of AAV2/9-GAD67-DIO-hM3D(Gq)-mCherry or AAV2/9-GAD67-DIO-mCherry into the CLA, and rAAV2/Retro-hSyn-CRE into the POR. (B) Representative confocal image showing injection site for hM3D(Gq) expression in the CLA with DAPI-counterstained. Scale bar, 100 µm. (C) Representative activity track maps of spontaneous activity from the indicated groups. (D) Total distance, central distance (%), and central time for OFT in mice (n = 6 mice per group). (E) Representative activity track maps of Morris water maze from the indicated groups. (F) Platform crossing times, effective distance, effective time, and effective distance (%) for Morris water maze in mice (n = 6 mice per group). (G) The print view and footfall patterns of the step sequence in a mouse that passes through the glass pane. CA, cruciate pattern (RF-LF-RH-LH). AA, alternate pattern (RF-RH-LF-LH). CB, cruciate pattern (LF-RF-LH-RH). (H) RH stand, RH stand index, RH mean intensity, Regularity index, Print Positions right paws, and Base of Support hind paws for CatWalk gait analysis in mice (n = 6 mice per group). Data are mean ± SEM; statistical analyses were conducted using unpaired *t* test. Statistical details are provided in Supplementary Table S1. CLA, claustrum. LF, left front. LH, left hind. POR, postrhinal cortex. RF, right front. RH, right hind.

**FIGURE S9** Chemogenetic inhibition of the CLA^VGluT2^-POR pathway in C57 mice. (A) Schematic of viral injection of AAV2/9-EF1a-DIO-hM4D(Gi)-mCherry or AAV2/9-EF1a-DIO-mCherry into the CLA, and rAAV2/Retro-VGluT2-SV40 NLS-Cre into the POR. (B) Representative confocal image showing injection site for hM4D(Gi) expression in the CLA with DAPI-counterstained. Scale bar, 100 µm. (C) Representative activity track maps of spontaneous activity from the indicated groups. (D) Total distance, central distance (%), and central time for OFT in mice (n = 6 mice per group). (E) Representative activity track maps of Morris water maze from the indicated groups. (F) Platform crossing times, effective distance, effective time, and effective distance (%) for Morris water maze in mice (n = 6 mice per group). (G) The print view and footfall patterns of the step sequence in a mouse that passes through the glass pane. CB, cruciate pattern (LF-RF-LH-RH). AA, alternate pattern (RF-RH-LF-LH). (H) RH stand, RH stand index, RH mean intensity, Regularity index, Print Positions right paws, and Base of Support hind paws for CatWalk gait analysis in mice (n = 6 mice per group). Data are mean ± SEM; statistical analyses were conducted using unpaired *t* test. Statistical details are provided in Supplementary Table S1. CLA, claustrum. LF, left front. LH, left hind. POR, postrhinal cortex. RF, right front. RH, right hind.

**FIGURE S10** Chemogenetic activation of the CLA^GAD67^-POR pathway in C57 mice. (A) Schematic of viral injection of AAV2/9-GAD67-DIO-hM3D(Gq)-mCherry or AAV2/9-GAD67-DIO-mCherry into the CLA, and rAAV2/Retro-hSyn-CRE into the POR. (B) Representative confocal image showing injection site for hM3D(Gq) expression in the CLA with DAPI-counterstained. Scale bar, 100 µm. (C) Representative activity track maps of spontaneous activity from the indicated groups. (D) Total distance, central distance (%), and central time for OFT in mice (n = 6 mice per group). (E) Representative activity track maps of Morris water maze from the indicated groups. (F) Platform crossing times, effective distance, effective time, and effective distance (%) for Morris water maze in mice (n = 6 mice per group). (G) The print view and footfall patterns of the step sequence in a mouse that passes through the glass pane. CB, cruciate pattern (LF-RF-LH-RH). AA, alternate pattern (RF-RH-LF-LH). AB, alternate pattern (LF-RH-RF-LH). CA, cruciate pattern (RF-LF-RH-LH). (H) RH stand, RH stand index, RH mean intensity, Regularity index, Print Positions right paws, and Base of Support hind paws for CatWalk gait analysis in mice (n = 6 mice per group). Data are mean ± SEM; statistical analyses were conducted using unpaired *t* test. Statistical details are provided in Supplementary Table S1. CLA, claustrum. LF, left front. LH, left hind. POR, postrhinal cortex. RF, right front. RH, right hind.

**FIGURE S11** Chemogenetic activation of the CLA^VGluT2^-POR pathway alleviates MS-like behaviors, experimental group received CNO + hM3Dq, control group received Saline + hM3Dq. (A) Schematic of viral injection of AAV2/9-EF1a-DIO-hM3D(Gq)-mCherry into the CLA, and rAAV2/Retro-VGluT2-SV40 NLS-Cre into the POR. (B) Representative confocal image showing injection site for hM3D(Gq) expression in the CLA with DAPI-counterstained. Scale bar, 100 µm. (C) Representative activity track maps of spontaneous activity from the indicated groups. (D) Total distance, central distance (%), and central time for OFT in mice (n = 6 mice per group). (E) Representative activity track maps of Morris water maze from the indicated groups. (F) Platform crossing times, effective distance, effective time, and effective distance (%) for Morris water maze in mice [n = 6 mice in Saline group, n = 10 mice in clozapine N-oxide (CNO) group]. (G) The print view and footfall patterns of the step sequence in a mouse that passes through the glass pane. AA, alternate pattern (RF-RH-LF-LH). AB, alternate pattern (LF-RH-RF-LH). CA, cruciate pattern (RF-LF-RH-LH). (H) RH stand, RH stand index, RH mean intensity, Regularity index, Print Positions right paws, and Base of Support hind paws for CatWalk gait analysis in mice (n = 6 mice in Saline group, n = 8 mice in CNO group). Data are mean ± SEM; statistical analyses were conducted using unpaired *t* test; **p <* 0.05, ***p <* 0.01, ****p <* 0.001. Statistical details are provided in Supplementary Table S1. CLA, claustrum. CNO, clozapine N-oxide. LF, left front. LH, left hind. POR, postrhinal cortex. RF, right front. RH, right hind.

**FIGURE S12** Chemogenetic inhibition of the CLA^GAD67^-POR pathway alleviates MS-like behaviors, experimental group received CNO + hM4Di, control group received Saline + hM4Di. (A) Schematic of viral injection of AAV2/9-GAD67-DIO-hM4D(Gi)-mCherry into the CLA, and rAAV2/Retro-hSyn-CRE into the POR. (B) Representative confocal image showing injection site for hM4D(Gi) expression in the CLA with DAPI-counterstained. Scale bar, 100 µm. (C) Representative activity track maps of spontaneous activity from the indicated groups. (D) Total distance, central distance (%), and central time for OFT in mice (n = 6 mice per group). (E) Representative activity track maps of Morris water maze from the indicated groups. (F) Platform crossing times, effective distance, effective time, and effective distance (%) for Morris water maze in mice (n = 6 mice in Saline group, n = 7 mice in CNO group). (G) The print view and footfall patterns of the step sequence in a mouse that passes through the glass pane. AA, alternate pattern (RF-RH-LF-LH). CB, cruciate pattern (LF-RF-LH-RH). AB, alternate pattern (LF-RH-RF-LH). (H) RH stand, RH stand index, RH mean intensity, Regularity index, Print Positions right paws, and Base of Support hind paws for CatWalk gait analysis in mice (n = 6 mice in Saline group, n = 8 mice in CNO group). Data are mean ± SEM; statistical analyses were conducted using unpaired *t* test; **p <* 0.05, ***p <* 0.01, ****p <* 0.001, *****p <* 0.0001. Statistical details are provided in Supplementary Table S1. CLA, claustrum. CNO, clozapine N-oxide. LF, left front. LH, left hind. POR, postrhinal cortex. RF, right front. RH, right hind.

**FIGURE S13** Representative injection sites at multiple anterior-posterior levels. (A-G) Different representative injection sites at multiple anterior-posterior levels in the CLA. Dashed lines delineate brain region boundaries. Scale bar, 400 µm. CLA, claustrum. LXN, latexin. POR, postrhinal cortex.

**FIGURE S14** FOS expression in the POR following chemogenetic manipulation of CLA-POR projections in MS mice. (A) Confocal images showing the FOS immunoreactive (green) in the POR in mCherry group in the CLA^VGluT2^-POR pathway. (B) Confocal images showing the FOS immunoreactive (green) in the POR in hM3Dq group in the CLA^VGluT2^-POR pathway. (C) The FOS^+^ neurons in the POR (n = 6 sections/3 mice per group). (D) Confocal images showing the FOS immunoreactive (green) in the POR in mCherry group in the CLA^GAD67^-POR pathway. (E) Confocal images showing the FOS immunoreactive (green) in the POR in hM4Di group in the CLA^GAD67^-POR pathway. (F) The FOS^+^ neurons in the POR (n = 6 sections/3 mice per group). White dashed line, the extent of the brain region. White solid lines, the magnified regions. Scale bar, 100 µm. Data are mean ± SEM; statistical analyses were conducted using unpaired *t* test; ***p <* 0.01, ****p <* 0.001. Statistical details are provided in Supplementary Table S1. CLA, claustrum. POR, postrhinal cortex.

**FIGURE S15** MS stimuli change excitability in the CLA-POR pathway. (A) Schematic of viral injection of AAV2/9-EF1a-DIO-GCaMP6s into the CLA, and AAV2/Retro-mVGLUT2-Cre into the POR, and optical fiber implantation in the CLA. (B) Fluorescence image showing viral expression and the trace of optical fiber implantation within the CLA. (C-E) Heatmap, averaged ΔF/F traces and area under the curve for calcium responses to control group and MS group (n = 8 per group, one data point per mouse). (F) Schematic of viral injection of AAV2/9-EF1a-DIO-GCaMP6s into the CLA, and AAV2/Retro-mVGAT1-Cre into the POR, and optical fiber implantation in the CLA. (G) Fluorescence image showing viral expression and the trace of optical fiber implantation within the CLA. (H-J) Heatmap, averaged ΔF/F traces and area under the curve for calcium responses to control group and MS group (n = 6 per group, one data point per mouse). Scale bar, 200 µm. Dashed lines delineate brain region boundaries; solid lines indicate fiber implantation boundaries. Data are mean ± SEM; statistical analyses were conducted using unpaired *t* test; **p <* 0.05. Statistical details are provided in Supplementary Table S1. Ctrl, control. MS, motion sickness. CLA, claustrum. POR, postrhinal cortex.

**TABLE S1** Detailed statistical results for all comparisons.

| Figure | Comparison | Test | Statistic | df | n | p value |
| --- | --- | --- | --- | --- | --- | --- |
| Figure 1B | Average kaolin intake (g) | Unpaired *t* test | t = 1.932 | 10 | n = 6 (Ctrl, MS group) | 0.0822 |
| Figure 1D | Total distance (mm) | Unpaired *t* test | t = 4.637 | 10 | n = 6 (Ctrl, MS group) | 0.0009 |
|  | Central distance (%) | Unpaired *t* test | t = 2.232 | 10 | n = 6 (Ctrl, MS group) | 0.0497 |
|  | Central time (s) | Unpaired *t* test | t = 2.891 | 10 | n = 6 (Ctrl, MS group) | 0.0161 |
| Figure 1F | Platform crossing times | Unpaired *t* test | t = 4.051 | 10 | n = 6 (Ctrl, MS group) | 0.0023 |
|  | Effective distance (cm) | Unpaired *t* test | t = 3.059 | 10 | n = 6 (Ctrl, MS group) | 0.0121 |
|  | Effective time (s) | Unpaired *t* test | t = 5.468 | 10 | n = 6 (Ctrl, MS group) | 0.0003 |
|  | Effective distance (%) | Unpaired *t* test | t = 3.698 | 10 | n = 6 (Ctrl, MS group) | 0.0041 |
| Figure 1H | RH_Stand (s) | Unpaired *t* test | t = 2.797 | 10 | n = 6 (Ctrl, MS group) | 0.0189 |
|  | RH_Stand Index | Unpaired *t* test | t = 3.584 | 10 | n = 6 (Ctrl, MS group) | 0.0050 |
|  | RH_Mean Intensity | Unpaired *t* test | t = 2.709 | 10 | n = 6 (Ctrl, MS group) | 0.0220 |
|  | Regularity index (%) | Unpaired *t* test | t = 2.890 | 10 | n = 6 (Ctrl, MS group) | 0.0161 |
|  | Print Position_Right Paws (cm) | Unpaired *t* test | t = 2.586 | 10 | n = 6 (Ctrl, MS group) | 0.0271 |
|  | Base of Support_Hind Paws (cm) | Unpaired *t* test | t = 6.868 | 10 | n = 6 (Ctrl, MS group) | < 0.0001 |
| Figure 2D | Double labeled neurons | Unpaired *t* test | t = 2.682 | 10 | n = 6 sections/3 mice (Ctrl, MS group) | 0.0230 |
| Figure 2F1 | Double labeled neurons | Unpaired *t* test | t = 2.306 | 10 | n = 6 sections/3 mice (Ctrl, MS group) | 0.0438 |
| Figure 2F2 | Triple labeled neurons | Unpaired *t* test | t = 2.707 | 10 | n = 6 sections/3 mice (Ctrl, MS group) | 0.0221 |
| Figure 2F3 | Double labeled neurons | Unpaired *t* test | t = 1.942 | 10 | n = 6 sections/3 mice (Ctrl, MS group) | 0.0808 |
| Figure 2H1 | Double labeled neurons | Unpaired *t* test | t = 2.195 | 10 | n = 6 sections/3 mice (Ctrl, MS group) | 0.0529 |
| Figure 2H2 | Triple labeled neurons | Unpaired *t* test | t = 2.059 | 10 | n = 6 sections/3 mice (Ctrl, MS group) | 0.0665 |
| Figure 2H3 | Double labeled neurons | Unpaired *t* test | t = 0.8682 | 10 | n = 6 sections/3 mice (Ctrl, MS group) | 0.4056 |
| Figure 2J1 | Double labeled neurons | Unpaired *t* test | t = 2.554 | 10 | n = 6 sections/3 mice (Ctrl, MS group) | 0.0286 |
| Figure 2J2 | Triple labeled neurons | Unpaired *t* test | t = 4.332 | 10 | n = 6 sections/3 mice (Ctrl, MS group) | 0.0015 |
| Figure 2J3 | Double labeled neurons | Unpaired *t* test | t = 0.7579 | 10 | n = 6 sections/3 mice (Ctrl, MS group) | 0.4660 |
| Figure 2L1 | Double labeled neurons | Unpaired *t* test | t = 2.508 | 10 | n = 6 sections/3 mice (Ctrl, MS group) | 0.0310 |
| Figure 2L2 | Triple labeled neurons | Unpaired *t* test | t = 2.410 | 10 | n = 6 sections/3 mice (Ctrl, MS group) | 0.0367 |
| Figure 2L3 | Double labeled neurons | Unpaired *t* test | t = 1.206 | 10 | n = 6 sections/3 mice (Ctrl, MS group) | 0.2556 |
| Figure 3D | Double labeled neurons | Unpaired *t* test | t = 3.049 | 10 | n = 6 sections/3 mice (Ctrl, MS group) | 0.0123 |
| Figure 3F | Triple labeled neurons | Unpaired *t* test | t = 2.469 | 10 | n = 6 sections/3 mice (Ctrl, MS group) | 0.0331 |
| Figure 3H | Triple labeled neurons | Unpaired *t* test | t = 2.677 | 10 | n = 6 sections/3 mice (Ctrl, MS group) | 0.0232 |
| Figure 3J | Triple labeled neurons | Unpaired *t* test | t = 1.000 | 10 | n = 6 sections/3 mice (Ctrl, MS group) | 0.3409 |
| Figure 3L | Triple labeled neurons | Unpaired *t* test | t = 1.534 | 10 | n = 6 sections/3 mice (Ctrl, MS group) | 0.1561 |
| Figure 5D | Total distance (mm) | Unpaired *t* test | t = 5.446 | 10 | n = 6 (mCherry, hM3Dq group) | 0.0003 |
|  | Central distance (%) | Unpaired *t* test | t = 2.702 | 10 | n = 6 (mCherry, hM3Dq group) | 0.0223 |
|  | Central time (s) | Unpaired *t* test | t = 2.598 | 10 | n = 6 (mCherry, hM3Dq group) | 0.0266 |
| Figure 5F | Platform crossing times | Unpaired *t* test | t = 3.244 | 16 | n = 8 (mCherry group)  n = 10 (hM3Dq group) | 0.0051 |
|  | Effective distance (cm) | Unpaired *t* test | t = 4.349 | 16 | n = 8 (mCherry group)  n = 10 (hM3Dq group) | 0.0005 |
|  | Effective time (s) | Unpaired *t* test | t = 4.084 | 16 | n = 8 (mCherry group)  n = 10 (hM3Dq group) | 0.0009 |
|  | Effective distance (%) | Unpaired *t* test | t = 4.719 | 16 | n = 8 (mCherry group)  n = 10 (hM3Dq group) | 0.0002 |
| Figure 5H | RH_Stand (s) | Unpaired *t* test | t = 0.07153 | 14 | n = 8 (mCherry, hM3Dq group) | 0.9440 |
|  | RH_Stand Index | Unpaired *t* test | t = 0.2537 | 14 | n = 8 (mCherry, hM3Dq group) | 0.8034 |
|  | RH_Mean Intensity | Unpaired *t* test | t = 0.5928 | 14 | n = 8 (mCherry, hM3Dq group) | 0.5628 |
|  | Regularity Index (%) | Unpaired *t* test | t = 1.180 | 14 | n = 8 (mCherry, hM3Dq group) | 0.2577 |
|  | Print Position_Right Paws (cm) | Unpaired *t* test | t = 0.5421 | 14 | n = 8 (mCherry, hM3Dq group) | 0.5963 |
|  | Base of Support_Hind Paws (cm) | Unpaired *t* test | t = 0.6060 | 14 | n = 8 (mCherry, hM3Dq group) | 0.5542 |
| Figure 6D | Total distance (mm) | Unpaired *t* test | t = 5.723 | 10 | n = 6 (mCherry, hM4Di group) | 0.0002 |
|  | Central distance (%) | Unpaired *t* test | t = 3.039 | 10 | n = 6 (mCherry, hM4Di group) | 0.0125 |
|  | Central time (s) | Unpaired *t* test | t = 2.375 | 10 | n = 6 (mCherry, hM4Di group) | 0.0390 |
| Figure 6F | Platform crossing times | Unpaired *t* test | t = 2.302 | 11 | n = 6 (mCherry group)  n = 7 (hM4Di group) | 0.0419 |
|  | Effective distance (cm) | Unpaired *t* test | t = 3.317 | 11 | n = 6 (mCherry group)  n = 7 (hM4Di group) | 0.0069 |
|  | Effective time (s) | Unpaired *t* test | t = 3.761 | 11 | n = 6 (mCherry group)  n = 7 (hM4Di group) | 0.0031 |
|  | Effective distance (%) | Unpaired *t* test | t = 3.608 | 11 | n = 6 (mCherry group)  n = 7 (hM4Di group) | 0.0041 |
| Figure 6H | RH_Stand (s) | Unpaired *t* test | t = 4.453 | 12 | n = 6 (mCherry group)  n = 8 (hM4Di group) | 0.0008 |
|  | RH_Stand Index | Unpaired *t* test | t = 3.907 | 12 | n = 6 (mCherry group)  n = 8 (hM4Di group) | 0.0004 |
|  | RH_Mean Intensity | Unpaired *t* test | t = 2.537 | 12 | n = 6 (mCherry group)  n = 8 (hM4Di group) | 0.0138 |
|  | Regularity Index (%) | Unpaired *t* test | t = 2.625 | 12 | n = 6 (mCherry group)  n = 8 (hM4Di group) | 0.0135 |
|  | Print Position_Right Paws (cm) | Unpaired *t* test | t = 2.204 | 12 | n = 6 (mCherry group)  n = 8 (hM4Di group) | 0.0478 |
|  | Base of Support_Hind Paws (cm) | Unpaired *t* test | t = 2.231 | 12 | n = 6 (mCherry group)  n = 8 (hM4Di group) | 0.0455 |
| Figure S2A | Average kaolin intake (g) | Unpaired *t* test | t = 1.419 | 10 | n = 6 (Ctrl, MS group) | 0.1862 |
| Figure S2C | Total distance (mm) | Unpaired *t* test | t = 0.6194 | 10 | n = 6 (Ctrl, MS group) | 0.5495 |
|  | Central distance (%) | Unpaired *t* test | t = 0.4960 | 10 | n = 6 (Ctrl, MS group) | 0.6306 |
|  | Central time (s) | Unpaired *t* test | t = 1.078 | 10 | n = 6 (Ctrl, MS group) | 0.3065 |
| Figure S2E | Platform Crossing times | Unpaired *t* test | t = 0.2911 | 10 | n = 6 (Ctrl, MS group) | 0.7769 |
|  | Effective distance (cm) | Unpaired *t* test | t = 0.4482 | 10 | n = 6 (Ctrl, MS group) | 0.6636 |
|  | Effective time (s) | Unpaired *t* test | t = 0.4948 | 10 | n = 6 (Ctrl, MS group) | 0.6315 |
|  | Effective distance (%) | Unpaired *t* test | t = 0.4081 | 10 | n = 6 (Ctrl, MS group) | 0.6918 |
| Figure S2G | RH_Stand (s) | Unpaired *t* test | t = 1.532 | 10 | n = 6 (Ctrl, MS group) | 0.1566 |
|  | RH_Stand Index | Unpaired *t* test | t = 0.7797 | 10 | n = 6 (Ctrl, MS group) | 0.4536 |
|  | RH_Mean Intensity | Unpaired *t* test | t = 0.2841 | 10 | n = 6 (Ctrl, MS group) | 0.7821 |
|  | Regularity index (%) | Unpaired *t* test | t = 0.03167 | 10 | n = 6 (Ctrl, MS group) | 0.9754 |
|  | Print Position_Right Paws (cm) | Unpaired *t* test | t = 0.8451 | 10 | n = 6 (Ctrl, MS group) | 0.4178 |
|  | Base of Support_Hind Paws (cm) | Unpaired *t* test | t = 0.2221 | 10 | n = 6 (Ctrl, MS group) | 0.8287 |
| Figure S3A | Total distance (cm) | Unpaired *t* test | t = 1.477 | 10 | n = 6 (Ctrl, MS group) | 0.1704 |
| Figure S3B | MS Index | Unpaired *t* test | t = 4.065 | 10 | n = 6 (Ctrl, MS group) | 0.0023 |
| Figure S3C | Total distance (cm) | Unpaired *t* test | t = 0.5482 | 10 | n = 6 (Ctrl, MS group) | 0.5956 |
| Figure S3D | MS Index | Unpaired *t* test | t = 2.150 | 10 | n = 6 (Ctrl, MS group) | 0.0570 |
| Figure S4D | FOS ^+^ Cells in MVePC | Two-way mixed-effects ANOVA | Row Factor: F (1.902, 9.510) = 82.35  Column Factor: F (1.000, 5.000) = 155.4  Row Factor x Column Factor: F (1.929, 9.643) = 38.26 | | n = 6 sections/3 mice (Ctrl, MS group) | 0.0001 (Day 0)  0.0059 (Day 1)  0.0847 (Day 3)  0.0865 (Day 5)  0.9801 (Day 7) |
| Figure S4F | FOS ^+^ Cells in CLA | Two-way mixed-effects ANOVA | Row Factor: F (2.111, 14.78) = 27.83  Column Factor: F (1.000, 7.000) = 30.36  Row Factor x Column Factor: F (2.666, 5.331) = 8.465 | | n = 6 sections/3 mice (Ctrl, MS group) | 0.0226 (Day 0)  0.1177 (Day 1)  0.2305 (Day 3)  > 0.9999 (Day 5)  0.9994 (Day 7) |
| Figure S4H | FOS ^+^ Cells in POR | Two-way mixed-effects ANOVA | Row Factor: F (2.032, 14.23) = 11.40  Column Factor: F (1.000, 7.000) = 2.052  Row Factor x Column Factor: F (2.186, 4.373) = 3.889 | | n = 6 sections/3 mice (Ctrl, MS group) | 0.0454 (Day 0)  0.8774 (Day 1)  0.9992 (Day 3)  0.9756 (Day 5)  > 0.9999 (Day 7) |
| Figure S7D | Total distance (mm) | Unpaired *t* test | t = 0.8835 | 10 | n = 6 (mCherry, hM4Di group) | 0.3977 |
|  | Central distance (%) | Unpaired *t* test | t = 0.3040 | 10 | n = 6 (mCherry, hM4Di group) | 0.7674 |
|  | Central time (s) | Unpaired *t* test | t = 0.1796 | 10 | n = 6 (mCherry, hM4Di group) | 0.8611 |
| Figure S7F | Platform crossing times | Unpaired *t* test | t = 1.466 | 12 | n = 8 (mCherry group)  n = 6 (hM4Di group) | 0.1683 |
|  | Effective distance (cm) | Unpaired *t* test | t = 1.044 | 12 | n = 8 (mCherry group)  n = 6 (hM4Di group) | 0.3170 |
|  | Effective time (s) | Unpaired *t* test | t = 1.753 | 12 | n = 8 (mCherry group)  n = 6 (hM4Di group) | 0.1050 |
|  | Effective distance (%) | Unpaired *t* test | t = 0.6530 | 12 | n = 8 (mCherry group)  n = 6 (hM4Di group) | 0.5261 |
| Figure S7H | RH_Stand (s) | Unpaired *t* test | t = 0.2557 | 14 | n = 8 (mCherry, hM4Di group) | 0.8019 |
|  | RH_Stand Index | Unpaired *t* test | t = 0.4339 | 14 | n = 8 (mCherry, hM4Di group) | 0.6710 |
|  | RH_Mean Intensity | Unpaired *t* test | t = 1.422 | 14 | n = 8 (mCherry, hM4Di group) | 0.1770 |
|  | Regularity Index (%) | Unpaired *t* test | t = 0.6366 | 14 | n = 8 (mCherry, hM4Di group) | 0.5346 |
|  | Print Position_Right Paws (cm) | Unpaired *t* test | t = 1.338 | 14 | n = 8 (mCherry, hM4Di group) | 0.2022 |
|  | Base of Support_Hind Paws (cm) | Unpaired *t* test | t = 1.184 | 14 | n = 8 (mCherry, hM4Di group) | 0.2561 |
| Figure S8D | Total distance (mm) | Unpaired *t* test | t = 0.8943 | 10 | n = 6 (mCherry, hM3Dq group) | 0.3922 |
|  | Central distance (%) | Unpaired *t* test | t = 0.05215 | 10 | n = 6 (mCherry, hM3Dq group) | 0.9594 |
|  | Central time (s) | Unpaired *t* test | t = 0.9242 | 10 | n = 6 (mCherry, hM3Dq group) | 0.3771 |
| Figure S8F | Platform crossing times | Unpaired *t* test | t = 1.449 | 10 | n = 6 (mCherry, hM3Dq group) | 0.1781 |
|  | Effective distance (cm) | Unpaired *t* test | t = 0.3745 | 10 | n = 6 (mCherry, hM3Dq group) | 0.7158 |
|  | Effective time (s) | Unpaired *t* test | t = 0.6705 | 10 | n = 6 (mCherry, hM3Dq group) | 0.5177 |
|  | Effective distance (%) | Unpaired *t* test | t = 0.01951 | 10 | n = 6 (mCherry, hM3Dq group) | 0.9848 |
| Figure S8H | RH_Stand (s) | Unpaired *t* test | t = 1.436 | 10 | n = 6 (mCherry, hM3Dq group) | 0.1814 |
|  | RH_Stand Index | Unpaired *t* test | t = 1.421 | 10 | n = 6 (mCherry, hM3Dq group) | 0.1858 |
|  | RH_Mean Intensity | Unpaired *t* test | t = 1.064 | 10 | n = 6 (mCherry, hM3Dq group) | 0.3125 |
|  | Regularity Index (%) | Unpaired *t* test | t = 1.786 | 10 | n = 6 (mCherry, hM3Dq group) | 0.1044 |
|  | Print Position_Right Paws (cm) | Unpaired *t* test | t = 1.851 | 10 | n = 6 (mCherry, hM3Dq group) | 0.0939 |
|  | Base of Support_Hind Paws (cm) | Unpaired *t* test | t = 1.701 | 10 | n = 6 (mCherry, hM3Dq group) | 0.1197 |
| Figure S9D | Total distance (mm) | Unpaired *t* test | t = 1.102 | 10 | n = 6 (mCherry, hM4Di group) | 0.2964 |
|  | Central distance (%) | Unpaired *t* test | t = 1.906 | 10 | n = 6 (mCherry, hM4Di group) | 0.0858 |
|  | Central time (s) | Unpaired *t* test | t = 0.5180 | 10 | n = 6 (mCherry, hM4Di group) | 0.6158 |
| Figure S9F | Platform crossing times | Unpaired *t* test | t = 0.07656 | 10 | n = 6 (mCherry, hM4Di group) | 0.9405 |
|  | Effective distance (cm) | Unpaired *t* test | t = 0.9619 | 10 | n = 6 (mCherry, hM4Di group) | 0.3588 |
|  | Effective time (s) | Unpaired *t* test | t = 1.567 | 10 | n = 6 (mCherry, hM4Di group) | 0.1481 |
|  | Effective distance (%) | Unpaired *t* test | t = 0.7738 | 10 | n = 6 (mCherry, hM4Di group) | 0.4569 |
| Figure S9H | RH_Stand (s) | Unpaired *t* test | t = 0.1946 | 10 | n = 6 (mCherry, hM4Di group) | 0.8496 |
|  | RH_Stand Index | Unpaired *t* test | t = 0.4744 | 10 | n = 6 (mCherry, hM4Di group) | 0.6454 |
|  | RH_Mean Intensity | Unpaired *t* test | t = 1.236 | 10 | n = 6 (mCherry, hM4Di group) | 0.2447 |
|  | Regularity Index (%) | Unpaired *t* test | t = 0.9711 | 10 | n = 6 (mCherry, hM4Di group) | 0.3544 |
|  | Print Position_Right Paws (cm) | Unpaired *t* test | t = 0.1237 | 10 | n = 6 (mCherry, hM4Di group) | 0.9040 |
|  | Base of Support_Hind Paws (cm) | Unpaired *t* test | t = 1.120 | 10 | n = 6 (mCherry, hM4Di group) | 0.2889 |
| Figure S10D | Total distance (mm) | Unpaired *t* test | t = 0.02337 | 10 | n = 6 (mCherry, hM3Dq group) | 0.9818 |
|  | Central distance (%) | Unpaired *t* test | t = 0.7449 | 10 | n = 6 (mCherry, hM3Dq group) | 0.4735 |
|  | Central time (s) | Unpaired *t* test | t = 1.831 | 10 | n = 6 (mCherry, hM3Dq group) | 0.0970 |
| Figure S10F | Platform crossing times | Unpaired *t* test | t = 1.574 | 10 | n = 6 (mCherry, hM3Dq group) | 0.1465 |
|  | Effective distance (cm) | Unpaired *t* test | t = 0.5000 | 10 | n = 6 (mCherry, hM3Dq group) | 0.6279 |
|  | Effective time (s) | Unpaired *t* test | t = 1.073 | 10 | n = 6 (mCherry, hM3Dq group) | 0.3083 |
|  | Effective distance (%) | Unpaired *t* test | t = 0.6070 | 10 | n = 6 (mCherry, hM3Dq group) | 0.5574 |
| Figure S10H | RH_Stand (s) | Unpaired *t* test | t = 0.4006 | 10 | n = 6 (mCherry, hM3Dq group) | 0.6971 |
|  | RH_Stand Index | Unpaired *t* test | t = 0.3288 | 10 | n = 6 (mCherry, hM3Dq group) | 0.7491 |
|  | RH_Mean Intensity | Unpaired *t* test | t = 0.7840 | 10 | n = 6 (mCherry, hM3Dq group) | 0.4512 |
|  | Regularity Index (%) | Unpaired *t* test | t = 0.6688 | 10 | n = 6 (mCherry, hM3Dq group) | 0.5188 |
|  | Print Position_Right Paws (cm) | Unpaired *t* test | t = 1.098 | 10 | n = 6 (mCherry, hM3Dq group) | 0.2978 |
|  | Base of Support_Hind Paws (cm) | Unpaired *t* test | t = 1.925 | 10 | n = 6 (mCherry, hM3Dq group) | 0.0832 |
| Figure S11D | Total distance (mm) | Unpaired *t* test | t = 5.349 | 10 | n = 6 (Saline, CNO group) | 0.0003 |
|  | Central distance (%) | Unpaired *t* test | t = 2.988 | 10 | n = 6 (Saline, CNO group) | 0.0136 |
|  | Central time (s) | Unpaired *t* test | t = 3.914 | 10 | n = 6 (Saline, CNO group) | 0.029 |
| Figure S11F | Platform crossing times | Unpaired *t* test | t = 3.994 | 14 | n = 6 (Saline group)  n = 10 (CNO group) | 0.0013 |
|  | Effective distance (cm) | Unpaired *t* test | t = 4.337 | 14 | n = 6 (Saline group)  n = 10 (CNO group) | 0.0007 |
|  | Effective time (s) | Unpaired *t* test | t = 3.210 | 14 | n = 6 (Saline group)  n = 10 (CNO group) | 0.0063 |
|  | Effective distance (%) | Unpaired *t* test | t = 4.193 | 14 | n = 6 (Saline group)  n = 10 (CNO group) | 0.0009 |
| Figure S11H | RH_Stand (s) | Unpaired *t* test | t = 0.5097 | 12 | n = 6 (Saline group)  n = 8 (CNO group) | 0.6195 |
|  | RH_Stand Index | Unpaired *t* test | t = 1.395 | 12 | n = 6 (Saline group)  n = 8 (CNO group) | 0.1882 |
|  | RH_Mean Intensity | Unpaired *t* test | t = 0.3338 | 12 | n = 6 (Saline group)  n = 8 (CNO group) | 0.7443 |
|  | Regularity Index (%) | Unpaired *t* test | t = 0.7649 | 12 | n = 6 (Saline group)  n = 8 (CNO group) | 0.4591 |
|  | Print Position_Right Paws (cm) | Unpaired *t* test | t = 0.3278 | 12 | n = 6 (Saline group)  n = 8 (CNO group) | 0.7487 |
|  | Base of Support_Hind Paws (cm) | Unpaired *t* test | t = 0.03951 | 12 | n = 6 (Saline group)  n = 8 (CNO group) | 0.9691 |
| Figure S12D | Total distance (mm) | Unpaired *t* test | t = 5.894 | 10 | n = 6 (Saline, CNO group) | 0.0002 |
|  | Central distance (%) | Unpaired *t* test | t = 2.367 | 10 | n = 6 (Saline, CNO group) | 0.0395 |
|  | Central time (s) | Unpaired *t* test | t = 2.999 | 10 | n = 6 (Saline, CNO group) | 0.0134 |
| Figure S12F | Platform crossing times | Unpaired *t* test | t = 2.484 | 11 | n = 6 (Saline group)  n = 7 (CNO group) | 0.0304 |
|  | Effective distance (cm) | Unpaired *t* test | t = 2.991 | 11 | n = 6 (Saline group)  n = 7 (CNO group) | 0.0123 |
|  | Effective time (s) | Unpaired *t* test | t = 3.723 | 11 | n = 6 (Saline group)  n = 7 (CNO group) | 0.0034 |
|  | Effective distance (%) | Unpaired *t* test | t = 4.003 | 11 | n = 6 (Saline group)  n = 7 (CNO group) | 0.0021 |
| Figure S12H | RH_Stand (s) | Unpaired *t* test | t = 5.965 | 12 | n = 6 (Saline group)  n = 8 (CNO group) | < 0.0001 |
|  | RH_Stand Index | Unpaired *t* test | t = 6.470 | 12 | n = 6 (Saline group)  n = 8 (CNO group) | < 0.0001 |
|  | RH_Mean Intensity | Unpaired *t* test | t = 2.819 | 12 | n = 6 (Saline group)  n = 8 (CNO group) | 0.0074 |
|  | Regularity Index (%) | Unpaired *t* test | t = 2.666 | 12 | n = 6 (Saline group)  n = 8 (CNO group) | 0.0123 |
|  | Print Position_Right Paws (cm) | Unpaired *t* test | t = 3.034 | 12 | n = 6 (Saline group)  n = 8 (CNO group) | 0.0104 |
|  | Base of Support_Hind Paws (cm) | Unpaired *t* test | t = 2.798 | 12 | n = 6 (Saline group)  n = 8 (CNO group) | 0.0161 |
| Figure S14C | FOS ^+^ neurons | Unpaired *t* test | t = 5.381 | 10 | n = 6 sections/3 mice (mCherry, hM3Dq group) | 0.0003 |
| Figure S14F | FOS ^+^ neurons | Unpaired *t* test | t = 3.597 | 10 | n = 6 sections/3 mice (mCherry, hM4Di group) | 0.0049 |
| Figure S15E | Area under curve | Unpaired *t* test | t = 2.366 | 14 | n = 8 (Ctrl, MS group) | 0.0329 |
| Figure S15J | Area under curve | Unpaired *t* test | t = 2.640 | 10 | n = 6 (Ctrl, MS group) | 0.0247 |

Abbreviation: CLA, claustrum. CNO, clozapine N-oxide. Ctrl, control. MS, motion sickness. MVePC, medial vestibular nucleus, parvicellular part. POR, postrhinal cortex. RH, right hind.
